# Realistic Modeling of the Electrocatalytic Process at Complex Solid‐Liquid Interface

**DOI:** 10.1002/advs.202303677

**Published:** 2023-09-25

**Authors:** Hongyan Zhao, Xinmao Lv, Yang‐Gang Wang

**Affiliations:** ^1^ Department of Chemistry and Guangdong Provincial Key Laboratory of Catalysis Southern University of Science and Technology Shenzhen Guangdong 518055 China

**Keywords:** Electrocatalysis, interfacial engineering, solid‐liquid interface, theoretical modeling

## Abstract

The rational design of electrocatalysis has emerged as one of the most thriving means for mitigating energy and environmental crises. The key to this effort is the understanding of the complex electrochemical interface, wherein the electrode potential as well as various internal factors such as H‐bond network, adsorbate coverage, and dynamic behavior of the interface collectively contribute to the electrocatalytic activity and selectivity. In this context, the authors have reviewed recent theoretical advances, and especially, the contributions to modeling the realistic electrocatalytic processes at complex electrochemical interfaces,  and illustrated the challenges and fundamental problems in this field. Specifically, the significance of the inclusion of explicit solvation and electrode potential as well as the strategies toward the design of highly efficient electrocatalysts are discussed. The structure‐activity relationships and their dynamic responses to the environment and catalytic functionality under working conditions are illustrated to be crucial factors for understanding the complexed interface and the electrocatalytic activities. It is hoped that this review can help spark new research passion and ultimately bring a step closer to a realistic and systematic modeling method for electrocatalysis.

## Graphical abstract

Advances, challenges, and perspectives in modeling the realistic electrocatalytic processes at complex solid‐liquid interfaces have been extensively reviewed and discussed. The significance of the inclusion of explicit solvation and electrode potential as well as the strategies toward the design of highly efficient electrocatalysts are crucial for rational design of electrocatalysts.

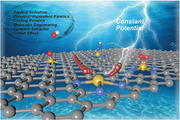

## 1 Introduction

The urgent need to address the severe environmental crisis and meet the ever‐expanding demand for sustainable energy has driven the development of renewable materials and techniques. One of the most promising avenues that can mitigate these environmental and energy dilemmas and rectify imbalances is electrochemistry,^[^ ^1^, ^2^, ^3^ ^]^ which has been recognized as one of the most thriving means for numerous catalytic processes, to name a few, CO_2_ reduction reaction (CO_2_RR),^[^ ^4^, ^5^, ^6^ ^]^ oxygen reduction reaction (ORR),^[^ ^7^, ^8^ ^]^ oxygen evolution reaction (OER),^[^ ^9^ ^]^ nitrogen reduction reaction (NRR),^[^ ^10^ ^]^ hydrogen evolution reaction (HER),^[^ ^11^ ^]^ etc. The success of these electrochemical processes has been evidenced to heavily depend on the screening and rational design of electrocatalysts^[^ ^12^ ^]^ and effective regulation of the electrochemical systems.^[^ ^13^, ^14^ ^]^ Therefore, advancing our atomistic understanding of the electrochemical environment is of paramount importance. Under realistic conditions, the elementary steps of electrochemical processes usually take place at the solid‐liquid interfaces and in particular solid‐water interfaces, where the reactants and intermediates not only interact with the catalyst substrate but also dynamically communicate with the solvent molecules. As a result, further developments in these areas require a comprehensive, atomic‐level understanding of many phenomena in chemistry, physics, and materials science and provide reasonable guidance for the rational design of electrochemical systems.

In electrochemistry, the development of electrode materials remains a significant research focus. Recent decades have witnessed great progress in the development of high‐performance electrocatalysts with superior activity, selectivity, and stability, among which, heterogeneous catalysts with atomically dispersed metal sites have gathered considerable attention since their innovative report in 1999.^[^ ^15^ ^]^ Subsequently, the terminology of single‐atom catalysts (SAC) was first proposed in 2011 based on isolated Pt_1_ dispersed on FeO*_x_* to highly oxidize CO,^[^ ^16^ ^]^ initiating an intensive exploration of the well‐defined active sites of electrocatalysts.^[^ ^17^, ^18^ ^]^ Downsizing metal sites to the atomic scale endows the catalysts with efficient atom utilization, a unique electronic configuration, and strong metal‐substrate interactions.^[^ ^19^ ^]^ Henceforth, the field of single‐atom catalysis has become one of the most active frontiers of heterogeneous catalysis.^[^ ^20^, ^21^, ^22^, ^23^ ^]^ Other heterogeneous catalysts, such as single‐cluster catalysts (SCC),^[^ ^24^, ^25^ ^]^ double‐atom catalysts (DAC),^[^ ^26^, ^27^ ^]^ and metal‐nitrogen catalysts (MNC),^[^ ^28^ ^]^ have also emerged from the philosophy of constructing well‐dispersed metals, forming a prosperous electrochemical community. In addition to developing the now‐popularized electrocatalysts, the rational design of active sites, including tuning the coordination environment and electronic structure, is a crucial measure to regulate the overall performance of the catalysts.^[^ ^29^ ^]^ Besides, metal electrodes have always been flourishing for their incontestable electrochemical performance.^[^ ^30^ ^]^

The solid‐liquid interface is a complex ensemble that requires comprehensive atomic insight to explore the elementary steps of the electrochemical reactions. Enormous efforts, both experimentally and computationally, have been devoted to this endeavor to probe the interfacial domain,^[^ ^31^ ^]^ which remains a challenging task, even for simple electrochemical systems. The inherent and decorated structures of active sites, along with their corresponding electronic nature are responsible for the electrocatalytic performance.^[^ ^32^ ^]^ The evolutional interface driven by the applied potential, giving birth to transient metastable states, should be taken into account when modeling the electrochemical process.^[^ ^33^ ^]^ Aside from the structure‐sensitivity character, the detailed properties of interfacial water, such as the physical orientation^[^ ^34^ ^]^ and hydrogen‐bonding network^[^ ^14^ ^]^ in electric double layers (EDL) have a significant impact on the electrochemical performances of electrode materials. Moreover, the cation‐dependent interfacial water structure, which yields different electric field strengths,^[^ ^35^, ^36^, ^37^ ^]^ adsorption rate,^[^ ^38^ ^]^ stability,^[^ ^39^ ^]^ and solvation pH,^[^ ^40^ ^]^ have also been investigated to understand the cation effect on the reaction kinetics. Other complexity arises from the descriptions of the molecular dynamics across the interface, which is highly potential‐dependent, and the associated thermodynamic modeling. Despite these challenges, it is necessary to investigate the condensed interface for a comprehensive understanding of electrochemical processes. However, experimental surface characterization methods have limited access to this interface, especially under opening electrochemical reaction conditions.

Although many advanced *operando* or in situ characterization techniques, such as scanning transmission electron microscopy (STEM),^[^ ^41^ ^]^ have been employed to probe the electrostatic potential distribution,^[^ ^42^ ^]^ ion distribution,^[^ ^43^ ^]^ and water orientation at the interfaces,^[^ ^34^, ^44^, ^45^, ^46^ ^]^ the extraordinary complexity of this solid‐liquid region is such that experimental techniques cannot readily characterize surface active sites and trace the progressive behavior at the atomic level. Additional insight is provided by theoretical studies to unravel the microscopic nature of these interfaces.^[^ ^47^ ^]^ From a computational modeling perspective, classical molecular dynamics (CMD) simulations have been the most widely employed technique to obtain a microscopic picture of solid‐liquid interfaces. Without elaborate thinking, we can cite a bunch of recent examples.^[^ ^48^ ^]^ Together with coarse continuum theoretical methods,^[^ ^49^ ^]^ such approaches are capable of connecting microscopic phenomena with meso/macroscopic ones, as they can cover extensive time and spatial scales, typically in nano magnitude. However, they heavily depend on the development of efficient, accurate, and universal empirical force fields and may not provide an exhaustive description of atomistic and electronic structure details, which are indispensable for describing the aspects of interfaces, such as electrochemical kinetics. This is where ab initio molecular dynamics (AIMD) simulations, with their nonempirical treatment of atomic interactions, come into play. AIMD simulations of heterogeneous interfaces represent a promising field, rapidly growing thanks to the advances in the hardware, availability through supercomputing centers, and the development of more efficient algorithms that exploit parallel programming.

In this thematic article, we present recent advances and opening challenges in the computational modeling of the solid‐liquid interface, reviewing how various effects are interpreted in the calculations and how they influence electrocatalytic activity and selectivity. First, we concentrate on practical atomistic descriptions of solid‐water interfaces, outlining current practices of first‐principles models for simulating heterogeneous electrochemistry, with an emphasis on our endeavors to realistically consider solvation shells and constant potential. We also present a unifying descriptor for thermodynamically and kinetically translating hydrogen evolution reaction (HER) activities on single‐atom catalysts (SACs). In the second session, we discuss critical aspects of the selected well‐defined cases. Critical issues here are the structures of the single‐atom (SA) sites, including the structure‐dependent electronic configuration of the active sites and its developing engineered strategy. Furthermore, we address surface dynamic behavior, interface water, (pseudo‐)adsorbates, and metal cations in the interfacial region. How does the applied potential or chemisorbed species drive the reversible or nonreversing restructuring of SAs and metal electrodes? How is the highly dynamic state of the water molecules and metal cations affected by the electrochemical condition? Finally, the upcoming specific challenges in realistic modeling of the solid‐water interface will be addressed, highlighting the integration of the theoretical and experimental methods in simulating heterogeneous electrocatalysis to advance their operation in rationally designing a more efficient interface. The field of electrochemical interface science is boundless, and the topics and cases discussed here are inevitably subjective. However, we anticipate that the subjects discussed will constitute important strides toward a more comprehensive understanding of electrochemical interfaces in the future.

## 2 Thermodynamic and Kinetic Modeling of the Electrocatalysis

In mechanistic investigations of electrochemistry, density functional theory (DFT) calculations have enabled us to comprehensively understand the reaction processes, which are far from a simple endeavor, but great development has been made in improving models. Recently, many great articles have exhaustively reviewed the computational modeling of electrocatalysis.^[^ ^50^, ^51^, ^52^ ^]^ Here, in the following section of this perspective, we specifically focus the subject on the major advances in simulation methods and our recent developments in describing the electrochemical interfaces and electrochemical reactions by utilizing first‐principle theory.

### 2.1 Computational Hydrogen Electrode Model

For electrocatalysis, one of the most widely employed models is the “computational hydrogen electrode” (CHE),^[^ ^53^ ^]^ allowing screen over substantial materials from a thermochemical view. In combination with DFT calculations, one can access adsorption energies and the free energy diagram of a specific reaction, taking into consideration of the zero‐point energy and entropy corrections.^[^ ^12^ ^]^ The CHE model regards the electrochemical solvated state of proton and electron as the equilibria of the nonelectrochemical state of hydrogen: 1/2H_2_(g) ⇆ H^+^ + e^˗^, allowing for the determination of the chemical potential for the proton‐electron pair. A typical case in point: the DFT‐predicted reactivities trend of MNCs toward OER is determined to be Ni > Co > Fe by employing the CHE model to consistently evaluate the Gibbs energies of all species involved in the electrochemical OER network while avoiding the explicit treatment of solvated protons and electrons in an alkaline environment.^[^ ^12^ ^]^ Effectively, the CHE‐based evaluation systems allow qualitatively establishing the correlation between the atomistic structure of the metal centers and the catalytic properties.

### 2.2 Explicit Solvation Shell

**Figure 1:**
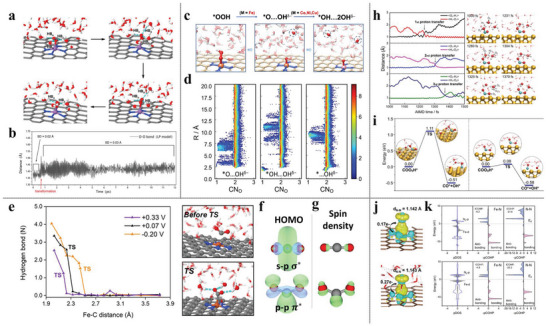
a) Selected snapshots from AIMD trajectories during the process of configurational transformation from end‐on *O_2_ to side‐on pattern with liquid water phase on FeNC. b) O–O bond length evolution during the AIMD simulation with explicit solvation. Reproduced with permission.^[^ ^57^ ^]^ Copyright 2020, American Chemical Society. c) Selected snapshots from AIMD trajectories show an unconventional dissociative ORR pathway that produces pseudo‐adsorbed hydroxide species. d) Probability density distribution of coordination number of O by H (CN_O_) in Z‐direction, from the three equilibrated AIMD trajectories of *O…OH^δ−^, *OH…OH^δ−^, and *…OH^δ−^, showing the spatial distribution of pseudo‐adsorbed OH. Reproduced with permission.^[^ ^13^ ^]^ Copyright 2022, Springer Nature. e) Statistic numbers of H‐bonds between the solvation water and CO_2_ reactant along the adsorption reaction coordinate at different potentials on FeNC and snapshots of the solvation environment around CO_2_ before and at TS. f) HOMO and g) spin density distribution of the CO^2−^ anion in a linear or bent configuration. Reproduced with permission.^[^ ^14^ ^]^ Copyright 2022, American Chemical Society. h) Evolution of the O‐H distances during proton transfer and the snapshots of the AIMD trajectories for the *COO_s_H to *COO_a_H species on Cu metal surfaces. i) Reaction free energy barrier comparison of CO formation form *COO_s_H and *COO_a_H. Reproduced with permission.^[^ ^58^ ^]^ Copyright 2022, American Chemical Society. j) Charge density difference and k) pDOS and pCOHP comparisons of N_2_ adsorption with (lower panel) and without (upper panel) water shell. Reproduced with permission.^[^ ^59^ ^]^ Copyright 2022, American Chemical Society.

The ignorance of explicit solvation shells in traditional free energy calculations cast doubt on mechanistic studies when we see a large discrepancy in solvation energies determined based on vacuum‐, implicit‐, explicit‐, or hybrid solvation models.^[^ ^54^, ^55^, ^56^ ^]^ Implicit continuum effects are accounted for by utilizing an electric field that modifies the adsorption energies^[^ ^39^ ^]^ while explicit electrolyte has been manifested to regulate or even subvert the reactivity and selectivity of metal electrodes. Recent AIMD and DFT simulations of ORR on MnNC with and without explicit solvation showed that the liquid water environment facilitates charge transfer from substrate to adsorbed *O_2_.^[^ ^57^ ^]^ Hydrogen bonding stabilization by neighboring water molecules converts the end‐on *O_2_ intermediate to a side‐on configuration (**Figure** **1**a) which is not the preferred geometry otherwise. This transformation with the elongation of the O–O bond distance takes place in a time scale of ≈0.5 ps and stays stable throughout the equilibrated trajectory (Figure 1b). The side‐on configuration strongly favors the dissociative pathway of ORR to form *O and the following protonation to form *OH and resulting in a more accurate estimation of the overpotential. By considering a fully explicit solvation scheme, Chen et al. have also discovered an unconventional ORR dissociative mechanism on FeNC electrocatalysts, in which the *OOH intermediate is found to spontaneously dissociate to form a “pseudo‐adsorbed” OH^δ−^ species which spontaneously migrates to the bulk solution phase through the hydrogen bond matrix and being spatially confined at a few water layers away from the catalyst surface (Figure 1c).^[^ ^13^ ^]^ Such pseudo‐adsorbed state in *O…OH^δ−^, *OH…OH^δ−^, and *…OH^δ−^ are dynamically confined in one of the shallow, thermal‐accessible local minimums on the flat free energy surface dominated by solvation (Figure 1d) so that the dissociated OH^δ−^ could respond to the redox event at the catalytic center in a coupled manner within a timescale less than 1 ps. Constrained MD investigations reveal that the H‐bonds between O in activated CO_2_ and the adjacent water molecules are rapidly formed once the TS is reached in the course of CO_2_ activation on FeNC (Figure 1e).^[^ ^14^ ^]^ Such configuration transforms originate from the HOMO‐LUMO crossover of the CO_2_ moiety, namely, the symmetric nonpolar s–p σ* HOMO state transforms to the distorted, highly polar p–p π* (Figure 1f), which is the original LUMO state, causing the spin density to redistribute to the terminal O atoms (Figure 1g), consequently contribute to a strong H‐bond acceptor. Through hydrogen bonding stabilization with an adjacent water environment, CO_2_ can be bent and adsorbed on NiNC otherwise will detach from the electrode.^[^ ^32^ ^]^ In the PCET steps of CO_2_RR, Yan et al. discover the hydrogenation of *CO_2_ is more favorable at the O site pointing to solvent (denoted as O_s_) on Cu electrocatalysts.^[^ ^58^ ^]^ However, AIMD simulations suggest an irreversible proton transfer from O_s_ to O_a_ (O_a_ refers to the O of *CO_2_ adsorbed on Cu surface (Figure 1h), via the local hydrogen bond network within a short timescale less than 1.5 ps, paving the way for the following dissociation step (Figure 1i). The solvation impact on the electronic configuration of the active center should be reckoned with. By comparing the N_2_ activation process with and without explicit consideration of solvent, Qian et al. confirm that the water environment can be responsible for the adsorption of N_2_ by facilitating the charge transfer from the metal center of FeNC to N_2_ (Figure 1j), furthermore, enhancing the interaction between both α and β pDOS of Fe's d orbitals with N_2_ p orbitals (Figure 1k).^[^ ^59^ ^]^

An extensive AIMD simulation has been conducted to examine the explicit solvation effects on the reaction energetics at a range of charged neutral metal‐water interfaces, and compared against the continuum solvent models such as CANDLE.^[^ ^60^ ^]^ Remarkable deviations of adsorption energies are identified between AIMD predictions and continuum solvation results, particularly for the strongly solvated adsorbates such as *OH and *OOH while no apparent superiority in stabilizing the adsorbates when adopting with and without implicit continuum solvation.^[^ ^56^ ^]^ The consideration of directional H‐bonds and steric water adsorption is evidenced to be essential for an accurate description of solvation at the metal‐water interfaces. In conclusion, all these challenges could contribute to the nontrivial character of explicit solvation shells. Notwithstanding, a coin has two sides. The incorporation of explicit solvation inevitably gives us expensive computations. Exploring more advanced algorithms to account for a greater number of explicit solvent environments without compromising ab initio accuracy is the subject of future investigations.

### 2.3 Scaling Relationship and the Volcano Plot

**Figure 2:**
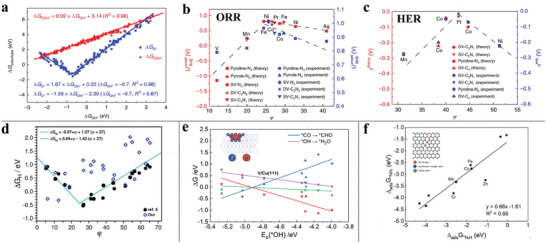
a) Adsorption‐free energies of *OOH and *O as a function of *OH on all studied MNCs. Theoretical and corresponding experimental onset potentials for b) ORR and c) HER versus the descriptor φ. Reproduced with permission.^[^ ^67^ ^]^ Copyright 2018, Springer Nature. d) The comparison results of adsorption‐free energies of H between PBE+U and the descriptor φ for MNCs. Reproduced under the terms of a CC‐BY license.^[^ ^70^ ^]^ Copyright 2022, The authors, published by ACS. e) Relationship between the reaction Gibbs free energy of the CO, CHO, OH, and H_2_O on TM_1_/Cu (111) as a function of *OH binding energy. Reproduced with permission.^[^ ^66^ ^]^ Copyright 2022, Tsinghua University Press. f) The linear relationship of the first and the last hydrogenation step of NRR on a series of dual‐atom catalysts by DFT calculations. Reproduced with permission.^[^ ^75^ ^]^ Copyright 2021, American Chemical Society.

When it comes to thermodynamics, the volcano plots, derived from the scaling relations^[^ ^61^ ^]^ that linearly correlate the surface binding energies of different adsorbates, including the transition state and reaction intermediate, are one of the most famous reactivity descriptors. The presence of such scaling correlation does conveniently compress Gibbs energy diagrams for numerous materials, ranging from transition metals,^[^ ^61^ ^]^ alloys,^[^ ^62^ ^]^ metal compounds,^[^ ^63^ ^]^ and molecular catalysts,^[^ ^64^ ^]^ and facilitates the simultaneous elucidation of the reactivity trends through the Sabatier‐type activity plots, which suggests that the optimal electrocatalyst candidates should possess an optimum binding strength between the adsorbates and surfaces, neither too weak that hinder activation nor too strong that prevent the desorption of the adsorbed species.^[^ ^65^ ^]^ Recent work by Jiang et al. investigated the relationship between the binding energy of one of the key species *OH (E_b, *OH_) and the ΔG of the potential determining steps (PDSs) in CO_2_RR to methane on various Cu‐based single transition metal atom alloys (TM_1_/Cu(111)) by DFT calculations.^[^ ^66^ ^]^ The inverted volcano‐shaped trend between *CO → *CHO (in blue) and *OH → *H_2_O (in purple) straightforwardly translates the optimal screening of the CO_2_RR electrocatalysts into finding materials with (E_b, *OH_) close to the volcano apex (**Figure** **2**e). The V/Cu (111), which is located nearest the volcano apex, exhibits the superior reactivity and selectivity toward CO_2_RR.

Xu et al. have demonstrated that the electrocatalytic activity of MNCs is strongly correlated with the local environment of the metal center, namely its coordination number (CN) and electronegativity, as well as the electronegativity of the nearest neighbor atoms.^[^ ^67^ ^]^ Specifically, the adsorption energetics of chemically similar adsorbates, such as *OOH, *O, and *OH involved in the ORR, OER, and HER, are found to be highly linearly correlated (Figure 2a). To address this, a universal descriptor φ is proposed by taking into account the valence electrons (θ_d_) of the metal center, combined with the electronegativity of the first coordinated nitrogen and second coordination carbon of the metal center. This descriptor, reputedly, is capable of interpreting the experimentally observed activity trends of the ORR (Figure 2b) and HER (Figure 2c), and outperforming other state‐of‐the‐art descriptors, such as orbital‐energy theory^[^ ^68^ ^]^ and the work function.^[^ ^69^ ^]^ This φ also works for SAC‐like metal‐macrocycle complexes and effectively reproduces the volcano relationships, which guides further rational optimization of the SACs. However, a subsequent theoretical investigation has revisited the HER activity and evaluated the performance of the φ descriptor on a series of MNCs. The results indicate that several factors, such as solvent effects, pH dependence, and even computational parameters of DFT methods, can lead to significant discrepancies in adsorption free energy of Δ*G*_H_ (Figure 2d).^[^ ^70^ ^]^ Furthermore, the revised HER volcano curve was obtained by linking microkinetics to the free‐energy diagrams, when the applied overpotential and kinetics are taken into account,^[^ ^71^, ^72^, ^73^ ^]^ highlighting the necessity to consider multiple factors when proposing universal descriptors from DFT screening.

Note, the screening strategy based on the scaling relations trivially assumes that all materials and facets follow the same reaction network,^[^ ^74^ ^]^ regardless of the reaction site, which inevitably poses a thermodynamic constraint on the studied catalysts and can lead to inappropriate simplification. Our evolving understanding toward scaling relation directs us to break or to circumvent it on account of the oversimplicity of the descriptor when screening or engineering potential electrocatalysts. For example, Chen et al.^[^ ^75^ ^]^ systematically explored ten heterogeneous double‐atom catalysts for electrocatalytic NRR by DFT calculations and found that the positive scaling relationship between the potential determining steps (PDSs) of the first and the last hydrogenation step of NRR, i.e., Δ_ads_ *G* ^*^N_2_H and Δ_ads_ *G* ^*^NH_2_ compromises the design for efficient NRR electrocatalysts (Figure 2f). As a result, the strategy to weaken the negative impact brought by the scaling relations is adopting optimal electrocatalysts with moderate Δ_ads_ *G* ^*^N_2_H and Δ_ads_ *G* ^*^NH_2_ (e.g., Cr_2_‐N_6_G, Mn_2_‐N_6_G, Fe_2_‐N_6_G, Co_2_‐N_6_G, and Zn_2_‐N_6_G), or to break it by the catalyst which favors a low Δ_ads_ *G* ^*^N_2_H and a high Δ_ads_ *G* ^*^NH_2_. In addition, synergistic effects in surfaces with various kinds of active sites, such as alloy, allow for circumventing the scaling relationships.^[^ ^76^, ^77^ ^]^ For instance, PtCu alloy could dramatically accomplish a better catalytic methanol electro‐oxidation reaction (MOR) performance compared with the pure Pt electrode.^[^ ^78^ ^]^ DFT calculations show alloying strategy not only reduces the free energy barriers for binding between *CO and *OH but also contributes to the water dissociation to produce more *OH and the resulting promoted anti‐poison reaction, arising from the dual‐sites effects. Other breaking strategies by multi‐active sites might be achieved using a binary oxide, or promoter‐functionalized surface.^[^ ^79^, ^80^ ^]^

**Figure 3:**
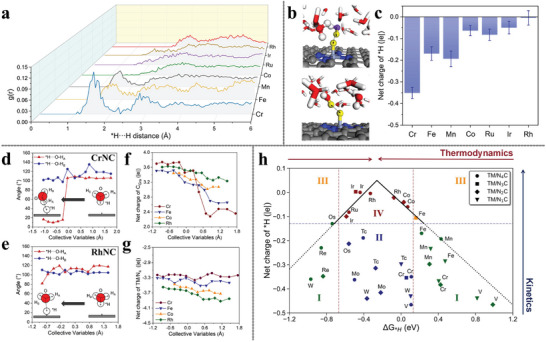
a) RDF analysis of directed H_2_O molecules around the *H in the sphere of *H···H distance on different MNCs. b) The illustration of the special *H…HOH moiety. c) The averaged Bader charges of *H on MNCs during AIMD simulations. Analysis of the angle between adsorbed hydrogen and O–H bond of water (*H···O–H) along the reaction coordinates of H adsorption on d) CrNC, and e) RhNC. Net charge evolution of f) graphene matrix and g) TM sites on different MNCs. h) Theoretical evaluation of the acidic HER activity on different MNCs. Reproduced with permission.^[^ ^85^ ^]^ Copyright 2023, American Chemical Society.

In addition, the volcano plots derived from the Sabatier principle are based on the underlying assumption of the Brønsted‐Evans‐Polanyi (BEP) relation, which assumes a correlation between the reaction energy and the corresponding barriers. However, it may not hold in cases where the reaction kinetics and thermodynamics are not strongly correlated or decorrelated. For instance, the nonmetallic nature of the SA site on MNCs shouldn't be overlooked in employing the traditional volcano plot to rationally design heterogeneous electrocatalysts for HER.^[^ ^81^ ^]^ From the Sabatier‐principle view, the MNCs with ΔG_*H_ value ≈ 0 show excellent HER rates.^[^ ^82^ ^]^ The CrNC and FeNC are predicted with the ΔG_*H_ in the range of −0.20 to 0.30 eV.^[^ ^83^ ^]^ However, the experimentally determined overpotential value of FeNC is ≈0.50 V.^[^ ^84^ ^]^ The substantial discrepancy appeals for an urgent re‐evaluation system on SA electrocatalysts. Our very recent theoretical work^[^ ^85^ ^]^ observes a strongly re‐orientated water configuration of *H···HOH motif (**Figure** **3**b) in which *H···O–H angle and distance are around to be ≈15° and ≈1.64 Å in AIMD simulations, evidenced by the pronounced peaks in the radial distribution function (RDF) analysis for CrNC, FeNC, and MnNC, located in the *H…H distance range of 1.0 Å–1.8 Å (Figure 3a). The corresponding hydride‐like feature in the three MNCs attributes the special neighboring water configuration of *H···HOH to the considerable accumulated electrons in *H (Figure 3c). Additionally, the abrupt configuration transition of the polarized electrolyte water (Figure 3d) in the course of H adsorption on CrNC by TI simulations echoes the charging behavior by the carbon matrix (Figure 3f) instead of the TM center (Figure 3g) while the gentle charging process on CoNC induces negligible structural disturbance of the surrounding environment. In the case of RhNC, the configurational change of neighboring H_2_O is not observed during the Volmer reaction (Figure 3e).

All the phenomena direct to the charge‐dipole interaction (CDI) between *H and neighboring H_2_O, which is quantified by $E_{CDI}=\frac{q_{i}\left(-q_{j}\right)e^{2}}{4\pi \varepsilon_{0}\left|r_{i}-r_{j}\right|}\;+\frac{q_{i}\left(q_{j}\right)e^{2}}{\pi \varepsilon_{0}\left|r_{i}+P_{j}-r_{j}\right|}$, leading to the E_CDI_ being briefly translated into the distance (r)‐ and net charge (q)‐dependent kinetics. A universal evaluation strategy toward the Volmer step, derived from the E_CDI_ kinetics in perspectives of coordinated N number, which arises the intrinsic variation of the SA per se identity,^[^ ^86^ ^]^ integrates not only the thermochemical information of ΔG_*H_ but, more importantly, the kinetics from the net charge of *H views (Figure 3h). This metric re‐inspects the kinetically unfavorable region II, where the conventional ‘volcano’ evaluation fails to describe, and inspires us with the interfacial interaction modulation tactics for the optimal design of the electrocatalysts. This very immediate contribution also emphasizes the critical role of the local solvent environment around the intermediates.`

### 2.4 Potential‐Dependent Electrocatalytic Reactivity

**Figure 4:**
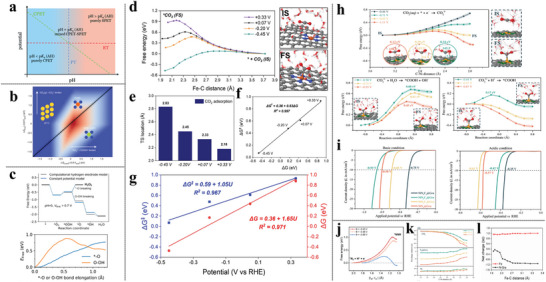
a) Pourbaix diagram showing the thermodynamic equilibria of the CPET, ET, and PT steps scale differently with pH. Reproduced with permission.^[^ ^91^ ^]^ Copyright 2016, the Royal Society of Chemistry. b) Rate map at −0.8 V_SHE_ and pH = 2 for CO_2_RR to CO across electrodes. Reproduced with permission.^[^ ^93^ ^]^ Copyright 2021, Springer Nature. c) The competing relation of the formation of H_2_O_2_ and H_2_O in the ORR process on CoNC in perspectives of thermodynamic (upper panel) and kinetic (lower panel) modeling. Reproduced with permission.^[^ ^96^ ^]^ Copyright 2021, American Chemical Society. d) Potential‐dependent free energy profiles of CO_2_ adsorption at the FeNC‐water interface. e) Location of transition states (TS) during adsorption at different potentials. The fitting linear relationship between f) ΔG and ΔG^‡^ and g) ΔG/ΔG^‡^ versus potential. Reproduced with permission.^[^ ^14^ ^]^ Copyright 2022, American Chemical Society. h) Potential‐dependent free energy profiles of CO_2_ adsorption (upper) and *COOH formation under basic (lower left panel) and acidic (lower right panel) environment on NiNC. i) LPD‐K predicted partial current densities for CO evolution in the basic (left) and acidic (right) phase. Reproduced with permission.^[^ ^88^ ^]^ Copyright 2022, American Chemical Society. j) Free energy profiles for *N_2_ hydrogenation to *NNH on FeNC. Reproduced with permission.^[^ ^59^ ^]^ Copyright 2022, American Chemical Society. Bader charge evolution in CO_2_ chemisorbing process on k) NiNC. Reproduced with permission.^[^ ^88^ ^]^ Copyright 2022, American Chemical Society. and l) FeNC. Reproduced with permission.^[^ ^14^ ^]^ Copyright 2022, American Chemical Society.

The scaling relation derived from the CHE model can facilitate the prediction of optimal electrocatalysts for certain electrocatalytic reactions with fewer independent variables to be considered. Nevertheless, the irrational generalization of proton‐coupled electron transfer (PCET) of all the elementary steps in CHE‐based models is not straightforwardly applicable to the decoupled proton‐electron transfer steps.^[^ ^87^ ^]^ Sequential proton‐electron transfers (SPET) have been prevailingly observed on MNCs^[^ ^14^, ^59^, ^88^ ^]^ and transition metals^[^ ^58^, ^89^, ^90^ ^]^ electrocatalysts. The mechanistic scenarios differ strongly across surfaces, applied potentials, and pH.^[^ ^91^, ^92^ ^]^ To illustrate this point, a complete picture of the activation stages in CO_2_RR on cobalt porphyrin electrocatalyst that allows for the transition between the sequential and concerted proton‐electron transfer mechanism is provided by Koper and colleagues.^[^ ^91^ ^]^ By quantifying the competition between PCET and SPET based on first‐principles calculations of acid‐base equilibrium constants, they rationalize the experimentally observed pH dependence of CO_2_RR activity (**Figure** **4**a). Recently, a Newns‐Andersen model has been reported to determine the rate‐determining step (RDS) of the CO_2_RR to CO on TMs, MNCs, and a supported phthalocyanine.^[^ ^93^ ^]^ Using this methodology, the electron transfer rate can be determined from the width of adsorbate‐induced projected density of states (pDOS). Combined with CHE‐predicted adsorption energies and pH‐dependent activity measurements, ET from TMs to CO_2_ to from *CO_2_ is determined to be RDS over active potential range, whereas either ET to form *CO_2_ or PCET to form *COOH is rate limiting on MNCs (Figure 4b).

In general, such correlations per se do not incorporate reaction kinetics from the effect of applied potential and arbitrarily regard the thermodynamic simulation as potential‐dependent kinetics, even without considering barriers which is an essential descriptor in the electrochemical process.^[^ ^87^, ^89^, ^94^, ^95^ ^]^ In the recently developed promising model of the “constant‐potential hybrid‐solvation dynamic model” (CP‐HS‐DM) by Liu et al,^[^ ^96^ ^]^ the competing relation of the formation of H_2_O_2_ and H_2_O in the course of ORR on CoNC was investigated. Specifically, they explored the reaction barriers of the key elementary steps of *OOH → *O + OH^−^ and *OOH + H_2_O → * + H_2_O_2_ + OH^−^, which demonstrated that the *−O bond breaking has a lower barrier than the *O−OH breaking. However, the thermodynamic information based on the CHE model gives a completely different conclusion that the strong thermodynamic preference for the O−OH bond breaking and the H_2_O formation (Figure 4c). In the kinetic ab initio modeling, another major open challenge of the interfaces is the determination of the driving force, i.e., the electrode potential. Existing methods include the ideal consideration employed in the CHE model. To be more specific, the potential treatment is based on the standard conditions (*T* = 298 K, pH = 0, *p* = 1 bar). *U* = 0 is defined as the electrode potential at which there is an equilibrium between an H^+^ and e^˗^ in an aqueous solution and a gaseous hydrogen (1/2H_2(g)_), as stated earlier.^[^ ^53^ ^]^ Furthermore, the chemical potential of the H^+^ and the e^−^ changes as the [H^+^] and the electrode potential varies.^[^ ^97^ ^]^ Despite the conquer of CHE in electrocatalysis, the explicit influence of varying electrode potentials on, for example, adsorption energies cannot be assessed given that varying electrode potentials lead to changes in the excess charge of the surface and the resulting electric fields.

In recent years, many theoretical efforts have been devoted to developing methods for investigating charged electrochemical systems.^[^ ^98^, ^99^, ^100^ ^]^ A predetermined number of electrons^[^ ^98^ ^]^ and counterions of potassium atoms^[^ ^99^ ^]^ are adopted to establish an approximate double layer. These methods are restrictive in that only an integral and constant number of excess electrons can be induced to the electrode surface, namely, that is under a canonical ensemble (NVT) possessing a varied Fermi level for different adsorbed species in each elementary step. However, since the configurational entropic contribution to the reaction kinetics plays a key role, many excellent studies have evidenced that the NVT MD simulation could yield more realistic solvation‐free energies and configurations while not compromising the accuracy too much from the potential variation along the reaction coordinate, based on the premise that multiple constant‐charge MDs with wide work function ranges to ensure proper equilibration and extensive sampling.^[^ ^14^, ^59^, ^88^, ^101^ ^]^ As the thematic issue presented here, AIMD simulations are still on the way to approaching a more realistic scenery of the electrochemical interfaces.

Despite the difficulties in the ab initio treatment of the electrochemical reaction barrier, we do need realistic considerations of kinetics for mechanistic understanding. Elementary‐step kinetics of the CO_2_RR mechanism on FeNC and NiNC with a sufficiently thick explicit water layer,^[^ ^14^, ^88^ ^]^ utilizing the aforementioned methods of surface charge tuning by introducing Na^+^ and K^+^ counterions, are systematically investigated by combining AIMD, constrained MD, and thermodynamic integration (TI). Free energy profiles are constructed at different potentials by TI on the equilibrated constrained AIMD trajectories (Figure 4d). It is observed that both the free energy (Δ*G*) and free energy barrier (ΔG^‡^) are linearly dependent on the electrode potentials (Figure 4g) in CO_2_ chemisorption courses. The total slope (*k* = 1.65) of the ΔG‐U relationship is not as simple as 1 eV/V on CO_2_ adsorption as suggested by the traditional CHE model, originating from the contribution of the potential‐dependent solvation effect is actually included during constrained MD simulation. Furthermore, the location of the transition state (TS) along the reaction coordinate shifts closer to the initial state (IS) as the applied potential decreases to a more negative value (Figure 4d). Likewise, this phenomenon is observed in the *CO formation process of *COOH + e^−^ → *CO + OH^−^ on the NiNC surface^[^ ^102^ ^]^ and the H adsorption step on MoS_2_^[^ ^103^ ^]^ by CI‐NEB methods.

Langmuir adsorption model‐derived potential‐dependent kinetics (LPD‐K)^[^ ^88^ ^]^ is proposed based on the assumption that CO_2_ activation should experience an adsorption/desorption equilibrium and the coverage obeys the Langmuir isotherm and can be determined by the applied potential. Using LPD‐K formulation, we investigate the potential‐dependent free energetics of the CO_2_RR on nitrogen coordinated single‐nickel atom catalysts in both acid and basic phase and conclude the RDS under acidic conditions is CO_2_ adsorption and under basic conditions, it is the first protonation step (Figure 4h). The calculated partial current densities and onset potentials with 10 mA cm^−2^ current density by LPD‐K (Figure 4i) are comparable with experimental values. Besides the CO_2_RR, the first hydrogenation step of *N_2_ to *NNH on FeNC shows potential‐dependent characters (Figure 4j).^[^ ^59^ ^]^ These findings benchmark that with sufficient free energy samplings, the NVT‐derived potential‐dependent kinetics could quantitatively match and well rationalize the experimental observed electrochemical activity on the MNC electrolyzer.

Using Bader charge analysis, the Fe and Ni centers, however, undergo negligible changes in the MNC motif and remain in their initial charge state. As expected, the charge source for activating CO_2_ is mainly contributed by the charged N‐doped graphene substrate (as an electron reservoir) (Figures 4k,l). As aforementioned, each constrained AIMD simulation is performed within the canonical ensemble with constant charge, which consequently comes with a shift in the work function along the reaction coordinate and the resultant varied electrode potential. Even with the adopted potential value (*U*_r_) of the studied elementary step being the average one of *U*_IS_ and *U*_FS_ (i.e., *U*_r_ = (*U*_IS_ + *U*_FS_)/2), strictly speaking, the methodology is not constant. Obtaining an accurate free energy landscape at a constant potential, i.e., doing sufficient sampling for both the configurational entropy and electronic contributions within the grand canonical ensemble (of electrons), has been remaining a challenging task.

### 2.5 Constant Potential Models

Theoretically, one of the most widely implemented first‐principles DFT methods to achieve the constant applied bias potential is fixing the work function or Fermi energy level of the electrode materials by adapting the number of electrons, n, to the applied constant potential, and then being referenced to an absolute potential of an electrode, such as SHE.^[^ ^104^ ^]^ The implementation of implicit solvation models enables continuous variation in the number of electrons of an electrolyte.^[^ ^105^, ^106^ ^]^ As a consequence, the grand free energy of an electrochemical system at a fixed bias is obtained by variationally optimizing n, and the generated charge will be balanced by ionic screening in the electrolyte. For example, Xiao et al. used the implicit CANDLE^[^ ^60^ ^]^ solvation model implemented in JDFT*x* ^[^ ^106^ ^]^ to investigate the atomistic mechanisms underlying electrochemical CORR on Cu (111)‐water interface, which including both the constant potential and solvation effect.^[^ ^105^ ^]^ These simulations successfully elucidated the competition among various pathways of CORR on Cu at different pH and accurately predicted the onset potentials from QM calculations, validating the particular combined consideration of constant potential and implicit electrolyte effect. It's worth noting that the introduction of n as an additional variable causes numerical instability for the self‐consistent field (SCF) method when solving the Kohn−Sham equations. The very recent publication by Xia et al. has formulated an efficient and robust fully converged constant‐potential (FCP) algorithm based on Newton's method and a polynomial fitting to accelerate the convergences, outperforming other algorithms, such as CG and BFGS.^[^ ^107^ ^]^ Benchmarked constant potential MD simulations of the first electrochemical hydrogenation step of CO catalyzed by the FeNC at *U* = −1.5 V revealed that the hydrogenation step is tend to occur via the CHO pathway, in agreement with previous experimental reports, exhibiting the accuracy of the grand canonical ensemble (µVT) modeling of electrochemical interfaces for the quantitative estimation of macroscopic electrochemical observation.

Although fractional charges, being introduced to achieve continuous work function and the corresponding controllable potential,^[^ ^100^ ^]^ offers the chance of electrochemical modeling at a constant potential since the Fermi level can be matched to the desired bias iteratively. The implicit solvation also brings about some extra errors and artifacts, for example, the induced balanced charge is unphysically dispersed in the electrolyte and renders the incapability of capturing the impact of double‐layer charging on reaction energetics. The use of work function as a descriptor of the driving force leads to cell size‐dependent functions of reaction energetics. In addition, the ignorance of explicit solvation shells, as aforementioned, will necessarily hinder our mechanistic knowledge as well.

An alternative to realizing the constant potential simulation is transforming the energetics based on the charged neutral model (CNM) to that on the constant potential model (CPM). Cell^[^ ^108^ ^]^ and charge^[^ ^109^ ^]^ extrapolation schemes based on fully explicit treatments of the electrolyte to deduce CPM, which are attainable for the evaluation of kinetic landscapes, both of which accommodate the effective surface‐charge density to appropriately depict the electrostatic effects of the double layer on reaction energetics^[^ ^110^ ^]^ on account of the description of the variations of the interfacial field local to the active region. However, recent studies^[^ ^52^ ^]^ have shed light on the complexity of the proposed cell‐extrapolation scheme, such as the inevitably prohibitive cost to apply to more complex electrochemical processes and larger‐size supercells. Due to the assumption that the “chemical” and electrostatic contributions to the energetics are separable (i.e., *E* = *E*_chem_ + *E*_el_), the charge‐extrapolation which defines that the interface functions as a simple capacitor, contradicts the behavior of the physical systems, especially in the case of 2‐dimensional materials.

**Figure 5:**
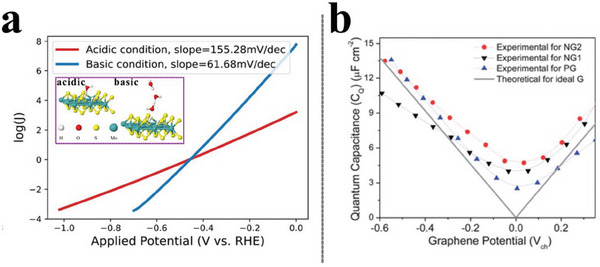
a) GCP‐K predicted Tafel plots for HER on MoS_2_ electrode in both acidic and basic conditions. Reproduced with permission.^[^ ^103^ ^]^ Copyright 2018, American Chemical Society. b) The experimental and theoretical quantum capacitance of pristine and N‐doped graphene as a function of electrode potential. Reproduced with permission.^[^ ^115^ ^]^ Copyright 2012, the Royal Society of Chemistry.

Grand canonical potential kinetics (GCP‐K)^[^ ^102^, ^103^ ^]^ are derived from minimizing the fixed‐charge free energy, F(n), to grand canonical, G (n, U) using Legendre transformation, allowing both the geometry of the transition states and the charge transfer from heterogeneous electrodes to adsorbates to change continuously along the reaction coordinate as the potential is changed. Using this GCP‐K predicted free energy, the hydrogen evolution reaction (HER) at a sulfur vacancy on the basal plane of MoS_2_ in both acidic and basic conditions is predicted.^[^ ^103^ ^]^ The higher activity of HER in basic originates from the TS of the Volmer step locates closer to FS compared with that in acid, leading to a more favorable Tafel slope of 60 mV/dec (**Figure** **5**a).

We notice that a quadradic term of F (*n*) is included in GCP‐K formulations, specifically, in which GCP (U) depends quadratically on the number of electrons, *n*, and the applied potential U, allowing a continuous description of the evolution of transition states. This quadratic grand canonical potential GCP (U) accounts for the change in capacitance as the potential changes. However, we cast doubt on the proposition because charge variation on 2D materials (such as the adopted representative cases of N‐doped graphene^[^ ^102^ ^]^ and MoS_2_^103^ in GCP‐K) can have a much stronger impact on the electrochemical reaction than the charge on 3D metals,^[^ ^111^ ^]^ without mentioning the widely employed fixed quantum capacitance value (*C*_d_) of 21 µF cm^−2^ of single‐layer pristine graphene deviate from experimental values.^[^ ^112^ ^]^ Way back in the decade, experimental^[^ ^113^, ^114^, ^115^ ^]^ and theoretical^[^ ^116^ ^]^ scientists have reported that the quantum capacitance of C_d_ is linearly dependent on the absolute potential for both pristine and N‐doped graphene electrodes (Figure 5b). The unconformity between the realistic potential‐dependent quantum capacitance and the adoptive constant one in the electrochemical elementary process will inevitably arise nonnegligible variations in accessing the reaction behaviors and stand in the way of the realistic interface. On the other hand, from the implicit solvation perspective, the adoption of continuum approaches in conjunction with explicit solvation shells in CP‐HS‐DM^[^ ^96^, ^117^ ^]^ offers a more realistic description of hydrogen bonding and solvation.

To the best of our knowledge, there was no theoretical continuous potential described elementary reaction thermodynamics and kinetics while the experimental investigations concentrate on the overall kinetic performance under continuous potential conditions. The linearity between the capacitance and potential, instead of the traditional consideration that the surface charge density linearly correlates with potential, calls for a pressing re‐examination of the potential‐dependence property. On the other hand, emerging hybrid explicit‐implicit approaches have the potential to advance the field of constant potential modeling, since they account for the solvation with cheap computational cost while retaining explicit electrolyte, representing a cutting‐edge method.^[^ ^110^ ^]^ However, developing effective implicit models that accurately describe the smooth transition of the permittivity between implicit and explicit solvation is another consequent issue.

## 3 Structure and Dynamics of Electrocatalysis at Complex Solid‐Liquid Interfaces

Distinct from the individual bulk media, solid‐liquid interfaces, and particularly solid‐water interfaces play significant roles in various territories of modern chemistry, such as heterogeneous electrocatalysis, and new materials design. To gain a deeper understanding and promote applications in a wider domain, a detailed comprehension of the interfaces including physical configuration, chemical and electronic structure and response to the environment at the atomistic level is a prerequisite to grasping the electrochemical process and its performance. In this section, we will specifically focus on the molecular representation of the electrode and water, and accordingly, examine the structure‐activity relationship. An elaborate microscopic description of solid‐liquid interfaces can be obtained from a theoretical point of view by AIMD simulations, which will be the primary approach used in this section. At this atomistic and molecular scale, the key questions to be addressed are,

- The molecular engineering at the interface from the perspective of active sites;
- How the chemical and physical configuration affects the electronic nature of the sites and the related electrochemical performance;
- The dynamic evolution of the potential‐ and pH‐dependent adsorbate coverage and the corresponding induced restructuring of electrodes and adaptive behavior of water molecules; what's more, we emphasize the dynamic behavior of reaction species, i.e., the dynamically confined ‘pseudo‐adsorption’ catalytic state;
- Considering the complexity of water solvation in realistic electrochemical systems, where ions are present at various concentrations, the effect of cations and interfacial pH must be incorporated since they are key elements that influence the relative organization of both the solid and the liquid as they come into contact continuously.

The structure‐performance relations of these active sites, their dynamic responses to the environment, and catalytic functionality under working conditions are crucial factors for understanding the interfaces.^[^ ^118^ ^]^

### 3.1 Engineering Electrocatalysts Via Structure Regulation

**Figure 6:**
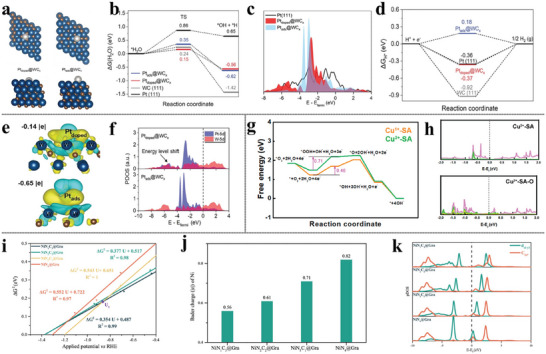
a) The theoretical structures of Pt_doped_@WC*_x_* and Pt_ads_@WC_x_. b) Calculated Gibbs free energy diagram for water dissociation on different electrocatalysts. c) Projected densities of states (PDOS) of Pt 5d orbitals of Pt (111), Pt_doped_@WC*_x_*, and Pt_ads_@WC*_x_*. d) Gibbs free energy of *H adsorption for different catalysts. e) The Bader charge analysis of the atomic Pt site. f) PDOS of Pt 5d orbitals and W 5d orbitals in the Pt_doped_@WC*_x_* and Pt_ads_@WC*_x_*. Reproduced with permission.^[^ ^120^ ^]^ Copyright 2022, Wiley‐VCH GmbH. g) Free energy diagram for ORR reaction pathways on Cu^1+^‐SA and Cu^2+^‐SA active sites in alkaline media. (pH = 13) h) PDOS overlap for Cu(I) and Cu(II) with *O atoms. Reproduced with permission.^[^ ^121^ ^]^ Copyright 2020, American Chemical Society. i) Linear correlation of the CO_2_ activation barrier, Δ*G* ^‡^, versus U_RHE_ on NiNx electrodes. j) Bader charge of Ni center for NiNx under PZC. k) PDOS of 3d*_x_*_2‑_ *_y_*_2_ orbitals for Ni center in all sites and C_2σ*_ orbitals of CO_2_. Reproduced with permission.^[^ ^88^ ^]^ Copyright 2022, American Chemical Society.

Heterogeneous electrocatalysts are intricate structures that are typically composed of several phases, generally, including an active catalytic phase and a supporting one with a conducting matrix onto which the nanoparticles of the active phase, such as transition metal atoms are embedded which not only stabilize but also collaborates with the active sites.^[^ ^119^ ^]^ Platinum‐based electrocatalysts occupy a pivotal position in the diverse catalytic process, but their scarcity and high cost have hindered large‐scale application. In our recent work, the crystalline lattice‐confined atomic Pt in tungsten carbide (Pt_doped_@WC*_X_*) exhibited competitive HER performance with Pt@C.^[^ ^120^ ^]^ Notably, DFT calculations revealed that the existence of the support phase of WC decreases the water dissociation energy barriers (**Figure** **6**b), leading to 40 times greater mass activity than that of the commercial Pt@C in alkaline phases. Charge density differences disclose that Pt_doped_@WC*_X_* exhibits near‐zero valence states (−0.14 |e|) and matched electronic structures as metallic Pt (Figure 6c), delivering similar catalytic behaviors when adsorbing H in the acidic phase (Figure 6d), which are much superior to the adsorbed Pt sites on WC (Pt_ads_@WC*_X_*), negatively charging by −0.65 |e| (Figure 6e). The different coordination pattern between the active site, Pt, and support matrix, WC, arises a huge deviation of the electronic configuration (Figure 6f), thus of the electrocatalytic activity. The novel electronic character of the SA site and the heterogeneity of its local environment cast doubt on mechanistic studies. The support of WC can also be a direct participant in the reaction. Despite both the water dissociation barriers on Pt_ads_@WC*_X_* (0.35 eV) and Pt_doped_@WC*_X_* (0.15 eV) are very low compared with that of metallic Pt (111) (0.86 eV) in the alkaline phase (Figure 6b), their dissociation properties are not identical, especially, regarding the adsorption site of produced *H. To be specific, on Pt_doped_@WC*_X_*, *H species adsorb directly at the Pt site, while on Pt_ads_@WC_X_, they adsorb at the W site, resulting in an additional 0.47 eV free energy compensation for *H migration from the W site to the Pt site during the subsequent H_2_ generation.

The character of structure‐sensitive performance is embodied in MNCs as well. Engineering the solid‐liquid interface by mixing the Cu salt precursor with varied urea content, a tailored coordination environment of the Cu (I) site with corresponding boosted ORR activity has been reported by Sun and coworkers.^[^ ^121^ ^]^ Theoretical calculations confirm the lower CNs with neighboring N of Cu feature a lower oxidation state, which facilitates the RDS of *O_2_ protonation to *OOH proceeding. The CHE‐predicted Δ*G* is lower on Cu (I) site by 0.25 eV compared to Cu (II) site (Figure 6g). The reactivity difference could be attributed to a less occupied O 2p‐d antibonding state in Cu (I), as revealed by the pDOS in Figure 6h. In the electrocatalytic conversion of CO_2_ to CO by NiN*_X_* (*_X_* = 1–4) moiety, our erewhile theoretical investigations by DFT‐based AIMD simulations confirm coordinately unsaturated nickel‐nitrogen sites on graphene (NiN_1_) achieve higher current density than others.^[^ ^88^ ^]^ Particularly, in the lower electrode potential regime of *U*_RHE_ > *U*_1_, NiN_1_, and NiN_2_ are more active than NiN_3_ and NiN_4_ towards CO_2_RR (Figure 6i), stemming from the lowest oxidation state of Ni in NiN_1_ (Figure 6j) and its occupation of the 3d*_x_*_2‐_ *_y_*_2_ orbital (HOMO) of the active center, Ni, which is more inclined to overlap with the C_2σ*_ (LUMO) orbital of CO_2_ (Figure 6k). Such catalyzing trends are ascribed to the charge capacity of Ni when the NiN_1_ structure has the lowest barriers for CO_2_RR and a high barrier for the competing reaction HER.^[^ ^32^ ^]^ In short, the modification of the metal SA with a well‐controlled coordination environment and the resulting modified low oxidation state can significantly contribute to the performance of SA sites.

**Figure 7:**
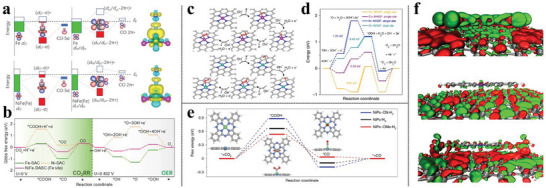
a) Schematic illustration of orbital interactions between adsorbed CO (5σ and 2π*) and 3d orbital (d_z2_, d_xz_/d_yz_) of Fe site in Fe‐SAC (upper) and NiFe‐DASC (lower). b) Calculated free energy diagrams for CO_2_RR and OER processes. Reproduced with permission.^[^ ^29^ ^]^ Copyright 2021, Springer Nature. c) Proposed reaction scheme of the single‐site (upper) and dual‐site (lower) mechanisms towards OER. d) Calculated free energy diagrams at 1.23 V for OER over FeNC, CoNC, and NiNC. Reproduced with permission.^[^ ^12^ ^]^ Copyright 2018, Springer Nature. e) Calculated free energy diagram of NiPc‐CN‐H_2_, NiPc‐H_2,_ and NiPc‐OMe‐H_2_ electrocatalysing CO_2_RR at −0.11 V_SHE_. Reproduced with permission.^[^ ^124^ ^]^ Copyright 2017, Springer Nature. f) Electron density difference plot showing charge transfer from graphene substrate to NiPc molecules. Reproduced with permission.^[^ ^126^ ^]^ Copyright 2021, American Chemical Society.

NiN_4_ moiety can serve as a structural promoter (Promoters are typically hetero‐atoms or hetero‐moieties that spread over the active surface to enhance catalytic activity or selectivity) of CoN_4_ and FeN_4_ sites in diatomic sites (DACs) to promote electrocatalytic performance.^[^ ^29^, ^122^ ^]^ Combined DFT and AIMD calculations reveal that the adjacent NiN_4_ site can effectively adjust the electronic localization of the proximity CoN_4_ site, promoting the *OH desorption and *H adsorption on the CoN_4_ site.^[^ ^120^ ^]^ Simultaneously, the orbital coupling between Fe and Ni lowers the molecular orbital energy levels of Fe and adsorbates (**Figure** **7**a) and weakens binding strength to the reaction intermediates, thus boosting CO_2_RR and OER performance (Figure 7b).^[^ ^29^ ^]^ It is worth noting that the active sites are not limited to the metal center for MNC electrocatalysts. Huang, Duan, and coworkers analyzed the relationship between the atomistic structure of the metal centers of Fe, Co, and Ni moieties and OER activities.^[^ ^12^ ^]^ It is theoretically predicted NiN_4_ exhibits the highest performance as revealed in Figure 7d, because of the collaborative participation of C atoms (a dual‐site mechanism, Figure 7c) in the OER process.

However, these 2D materials suffer from the instability of the sintering of the nanoparticles of the active component with a low surface area.^[^ ^123^ ^]^ Additionally, the uncontrollable doping environment leads to difficulties in defining the local electronic structure of the SA sites. To address these issues, researchers have explored and designed a new class of molecular electrocatalysts. In 2017, ZHANG et al. reported the noncovalent immobilization of molecularly engineered cobalt phthalocyanine on carbon nanotubes (CoPc@CNTs), achieving superior faradaic efficiency (FE) for CO_2_RR to CO.^[^ ^124^ ^]^ The structural uniformity and tunability, as well as the π–π interaction that preserves the molecular integrity and fine‐tunes the electronic structure of the active sites. Subsequently, a series of further engineered NiPc@CNTs achieved unit conversion to CO with high current density, with tetramethoxy substitution as an electron‐donating group (EDG) improving the stability of NiPc, and cyano group (–CN) as an electron‐withdrawing group (EWG) destabilizing the catalysts in long‐term electrocatalysis.^[^ ^125^ ^]^ Atomic charge analysis revealed that the introduced ‐OME to the NiPc enriched the electron density of the NiN4 moiety, resulting in strengthened COOH binding under the CHE scheme, while weakened CO adsorption prevented the poisoning of the active site (Figure 7e). Furthermore, the Ni‐N bond was strengthened by the more electron‐rich metal center, which protected the pristine NiN4 moiety from dissolution and the resultant deactivation.^[^ ^126^ ^]^ In our recent theoretical study, the interaction effect and charge transfer kinetics between NiPc molecules and the nanocarbon support were investigated using the electron density difference map.^[^ ^126^ ^]^ The enhanced polarization of NiPc‐CN and the resultant stronger interaction with the graphene substrate (Figure 7f, upper panel) facilitates the charge transfer across the interface, which is significant in electrocatalysis but is often neglected in computational modeling. These findings open up an additional dimension in molecular design.

### 3.2 Dynamics of the Solid‐Liquid Interfaces

Hitherto, a plethora of experimental and theoretical reports have been focused on the various electrocatalysts model to investigate their mechanistic behavior and reactivity trends. However, solely understanding the interface in its resting state may not always be sufficient to rationalize the electrocatalytic performance. Our findings suggest that Our obtained knowledge and findings suggest that our scope should not be confined to stationary form.^[^ ^17^, ^18^ ^]^ During catalytic conditions, however, the surface of heterogeneous electrodes, together with the water molecules are subject to surrounding mediums, i.e., the electrochemical potential, pH, temperature, and the coverage of adsorbates, which could render the catalytic electrode dynamic, make the surface reconstruction,^[^ ^127^, ^128^ ^]^ and induce transient metastable surface species.^[^ ^75^, ^129^ ^]^ Capturing and harnessing the dynamic distribution of the direct participating species at different physical scales in the electrochemical interface is of importance when modeling the active interface. This section specifically highlights this particular research field: the dynamic behavior of the interface, which incorporates the leaching behavior of the SA sites and surface restructuring of the metal electrode; the adaptive coordination and orientation of the water molecules; and the dynamically confined ‘pseudo‐adsorption’ catalytic process. Advancements in theoretical understanding make it possible to harness the dynamic interface to a greater extent and in a more systematic way.

#### 3.2.1 The Leaching of the SA Sites

The electrocatalytic performances of electrode materials are inherently determined by the number and structure of the active sites, which are critical and yet less controllable due to the complexity involved in the electrocatalytic process. This complexity is further compounded by the fact that different preparation methods, such as temperature and atmosphere, can result in diverse active structures.^[^ ^130^, ^131^, ^132^ ^]^

For example, the genuine site structure for ORR in the case of CuNC electrocatalyst has ever been claimed to be CuN_2_,^[^ ^133^, ^134^ ^]^ CuN_3_,^[^ ^135^ ^]^ and CuN_4_,^[^ ^136^ ^]^ without a consensus arise not only from the different preparation methods, which may result in diverse active structures but also from the challenge that the transient lifetime of SA sites and the limited spatial resolution makes it difficult to probe directly in atomistic dimension by experimental methods under operating conditions. In fact, the dynamic evolution of the active site is ubiquitous in catalysis.^[^ ^137^, ^138^ ^]^ Wang et al. have studied the reaction mechanism of CO oxidation on ceria‐supported Au catalysts static using DFT calculations and AIMD simulations.^[^ ^18^ ^]^ An initiative dynamic single‐atom catalytic mechanism was identified, in which CO adsorption engenders an isolated cationic Au^+^˗CO unit's formation first, facilitating the subsequent CeO_2_ reduction and CO oxidation. After the catalytic cycle, the Au single atom is reintegrated into the Au nanoparticle, indicating that the actual catalytic single‐atom species only exists as a transient under operating conditions which may not necessarily be easy to capture ex situ. Besides, the adsorbate‐induced novel dynamism is highly relevant to both the temperature and oxygen partial pressure.^[^ ^17^ ^]^

**Figure 8:**
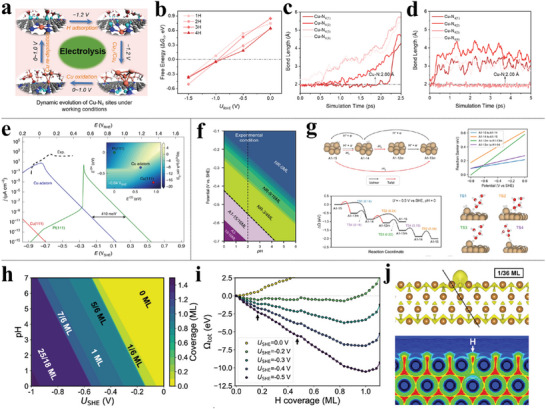
a) Illustration of the dynamic reversible transformation between the CuNC and Cu_4_ clusters under working conditions. b) Calculated free energy (ΔG_H_) of the 1^st^, 2^nd^, 3^rd^, and 4^th^ H adsorption under different potentials. Cu‐N bond lengths evolution for c) the complete SA leaching and d) the incomplete one. Reproduced with permission.^[^ ^117^ ^]^ Copyright 2022, American Chemical Society. e) Calculated current densities for the Pt (111), Cu (111), and the undercoordinated Cu adatom active site motif generated at the restructuring Cu electrode. Reproduced with permission.^[^ ^152^ ^]^ Copyright 2020, Springer Nature. f) Calculated surface Pourbaix diagram showing potential and pH dependence of the Cu (111) surface state. g) Reactive evolution of the A1‐15 structure. Reproduced with permission.^[^ ^127^ ^]^ Copyright 2023, Wiley‐VCH GmbH. h) Calculated surface Pourbaix diagram showing potential and pH dependence of the Cu (100) surface state. i) Total grand canonical free energy as a function of H coverage at different electrode potentials. j) Color‐filled contour map of Cu (100) with 1 H showing a weakening of the Cu‐Cu bond between the top layer and the sublayer. Reproduced with permission.^[^ ^128^ ^]^ Copyright 2022, American Chemical Society.

During the electrochemical reactions catalyzed by MNC materials, both adsorbates and applied potentials may drive the dynamic evolution of the SAs.^[^ ^139^, ^140^, ^141^ ^]^ ZHAO et al. utilized CP‐HS‐DM^[^ ^96^ ^]^ to investigate the dynamic progression of CuNC under reducing potentials.^[^ ^117^ ^]^ The highly thermodynamically favored H adsorption on N sites in the large potential regime of *U*_RHE_ < −1.0 V, as shown in **Figure** **8**b, is the direct driving force of the notorious restructuring phenomenon of CuNC. After adsorbing one or two H^+^, two transient states of Cu, with the incomplete leaching being tethered to one N atom (Figure 8c) and the complete leaching dissolved in water (Figure 8d), have been identified at a dynamic electrochemical interface by MD simulations within a timescale <0.5 ps. The reversible transformation between Cu SA and Cu_3/4_ clusters is further declared by the MD observations, in which the two leached copper atoms approach one another and concomitant by the elongation of the Cu–N bond and in the thermodynamically favorable dispersion process, the corresponding Cu–Cu bond elongation is identified. Be that as it may, subject to the expensive computational cost, the following aggregation of the simple Cu_4_ clusters and the reverse fragmentation process is not completely depicted. This work highlights that the adsorption of H, depending on the potentials, is a crucial driving force for Cu SA to Cu clusters, while the reverse process is dominated by Cu oxidated by OH radicals. As far as we concerned, this work is far from satisfactory on account of the discrete potential simulation and arbitrary introduction of the He atom and OH radicals. In addition, they unreasonably concluded the processes of SA aggregation, CO_2_RR, and decomposition of Cu_4_ clusters are sequential (Figure 8a). The considered reducing potential for the CuNC reconstruction, −1.2 V, is in the CO_2_RR active region^[^ ^142^, ^143^ ^]^ so that the evolving Cu and the resulting undercoordinated configuration are never static but accompanied by electrocatalyzing the CO_2_RR. As many literatures pointed out that the transient low‐coordinated Cu SA sites have a much better performance toward CO_2_RR.^[^ ^144^, ^145^ ^]^ As a result, we wonder about the genuine active site for the presentative high electrocatalytic CO_2_RR on CuNC under operating conditions. In addition to theoretical simulations, numerous experimental explorations identified the reduction of Cu^2+^ to Cu^+^ and Cu^0^ and the subsequent aggregation of Cu^0^ single atoms occurs concurrently with the enhancement of the NH_3_ production rate and ORR, both of them are driven by the applied potential switching from 0.00 to −1.00 V versus RHE.^[^ ^10^, ^33^ ^]^ By combining operando XANES spectroscopy and DFT calculations, the potential driven dynamic evolution of CuNC from Cu^2+^‐N_4_ to Cu^+^‐N_3_ and further to HO‐Cu^+^‐N_2_ has been unambiguously identified.^[^ ^33^ ^]^

This transformation of the Cu SA site is ascribed to the fact that the more negative potential drive more electron aggregate on the catalyst surface, promoting the adsorption of H^+^, which is a prerequisite driving force for the leaching of Cu single atoms from the catalyst surface and transforming into Cu small agglomerates. From the micro‐electronic perspective, the fully occupied 3d orbitals of Cu atom^[^ ^146^ ^]^ and the consequential weak Cu‐N bond, induced Cu and N sites tended to react with H^+^ and resulting in the breakage of the Cu‐N bond. This is the underlying reason that the leading actor in the leaching phenomenon is notorious copper. However, NiNC,^[^ ^147^ ^]^ CoNC,^[^ ^13^ ^]^ and FeNC^[^ ^13^, ^148^ ^]^ echoed.

#### 3.2.2 Surface Restructuring

The underlying explanation here is that, under opening electrochemical conditions, the adsorbates engender the metal electrode to undergo a structural transformation, sometimes reversible, of liberating from the substrate and evolving into a more active phase, which includes but is not limited to nanocluster,^[^ ^127^, ^149^, ^150^ ^]^ transitional surface phase,^[^ ^151^ ^]^ and undercoordinated structure.^[^ ^152^ ^]^ In situ EC‐STM techniques monitored reversible CO‐adsorption‐induced local morphological changes of Cu (111) with flat terraces and smooth edges under a reducing potential of −0.85 V_SHE_ to a threadlike Cu nanostructure and small adatom islands when potential increases from −0.85 to −0.45 V_SHE_.^[^ ^152^ ^]^ Theoretical microkinetic simulations evidence that the metastable Cu adatom is much more active than Cu (111) surface in CO oxidation and even comparable with Pt (111) (Figure 8e), benefiting from the enhanced electronic back donation from Cu 5d electrons to CO. This structure dynamism is not observed in absence of CO, since the Cu adatom tends to aggregate if not stabilized by CO, which is identified by the very newly released work of Cu (111) electrocatalyzing HER in acidic electrolyte.^[^ ^127^ ^]^ The strong H adsorption and the resulting high coverage under reductive potential engender the Cu (111) configurational transformation from a pristine metal surface to an organized (4×4) superstructure of Cu adatoms with 12 H adsorbates in the unit cell, combining triangular and square arrangements, which is captured by *operando* ECSTM at the potential range of −0.25 to −0.27 V versus SHE at pH = 2. Cheng et al. utilize global optimization algorithms and GC‐DFT calculations to efficiently identify the active potential window for HER on the Cu (111) surface in an acidic solution lies in the stable potential range of −0.42 V to −0.58 V of the formed low coordination Cu adatoms (A1‐15) (Figure 8f).^[^ ^127^ ^]^ The restructured A1‐15 Cu adatoms with H show much better electro‐performance with lower reaction barriers toward HER than pure Cu (111) metal surface (Figure 8g).

In acidic media, the adsorbate *H can not only drive the Cu adatoms evolving but also induce dramatic restructuring of the formation of stripes on Cu (100) metal surfaces at ≈−0.4 V, as observed by in situ STM.^[^ ^153^ ^]^ The enormous chemical space of Cu (100) spanned in structural and configurational dimensions under varying applied potentials is explored efficiently by global optimizations using GCGA, which not only reveals the origin but the structure evolution of the reported stripe pattern.^[^ ^128^ ^]^ The row‐shifting, triggered by H adsorption and negative potential (Figure 8h), is promoted thermodynamically and kinetically as potential decreases and H coverage (θ_*H_) increases (Figure 8i), originating electronically from the weakening of Cu–Cu bonds between the top‐ and sub‐layer by the H migration by chemical bonding analysis (Figure 8j). The detailed stripe formation pathway with multi‐intermediate stages as H coverage varies is uncovered atomically.

#### 3.2.3 The Adaptive Response of Electrolytes and Reaction Species

**Figure 9:**
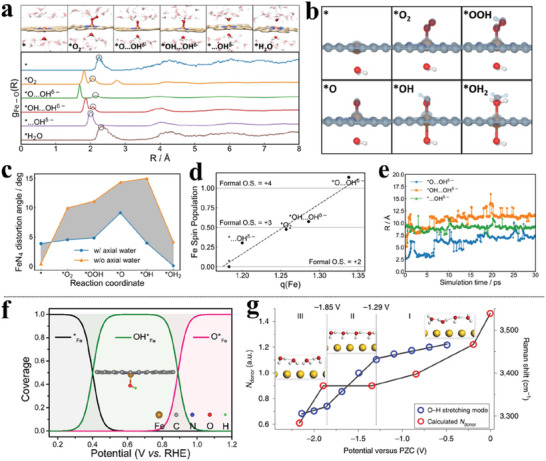
a) The radial distribution function g(r) of the backside axial water on FeNC in different reaction intermediates with representative snapshots labeled on the top. b) Overlapped geometries of gas phase reaction intermediate with (colored) or without (transparent blue) backside axial water ligand. c) The evolution of FeNC distortion angle along the reaction coordinate for gas phase reaction intermediates with (blue lines) or without (orange lines) backside water ligand. The shaded area represents the difference of FeNC distortion angle in the two cases. d) The scatter plot of Mulliken spin population versus the Bader charges on Fe along the reaction coordinate. e) The z coordinate of the O in dissociated OH^δ−^ relative to that of the Fe single site (denoted as R) in *O…OH^δ−^, *OH…OH^δ−^, and *…OH^δ−^ intermediates. Reproduced with permission.^[^ ^13^ ^]^ Copyright 2022, Springer Nature. f) Coverages of the most abundant ORR intermediates on the SA Fe site of the FeNC center as a function of U. Reproduced with permission.^[^ ^148^ ^]^ Copyright 2019, American Chemical Society. g) Calculated number of hydrogen‐bond donors (N_donor_) of interfacial water (red circles) as a function of potential, in comparison to the experimental Raman frequency ν_O–H_ (blue circle). Reproduced with permission.^[^ ^34^ ^]^ Copyright 2019, Springer Nature.

Apart from the dynamic behavior of the electrode, water molecules are sensitive to the reaction conditions. The dynamic coordination behaviors of the SA site are reported on FeNC and MnNC in the course of ORR.^[^ ^13^, ^148^ ^]^ Chen et al. carried out AIMD on FeNC with a fully explicit solvation shell to discover solvent water can dynamically coordinate to the Fe center in the backside axial position to adapt the binding strength and coordination configuration as the reaction proceeds, to module the ORR energetics and structural stability of the MNC moiety by varying the Fe‐O_axial_ bond lengths (**Figure** **9**a).^[^ ^13^ ^]^ In particular, the high potential‐induced leaching observation is a big concern of the stability of MNCs in long‐term operation.^[^ ^147^, ^154^ ^]^ However, the backside axial water stabilizes the Fe center through the octahedral configuration by alleviating the out‐plane distortion of SA sites. Resorting to a simplified gas phase model with or without the backside axial water ligand, a significant geometric difference in the FeN_4_ moiety is observed. (Figure 9b) For instance, in the *OH intermediate without backside water, the Fe center is dragged out of the carbon matrix plane by the hydroxyl dramatically to form a square pyramidal configuration, while in the *OH with backside axial water the Fe is relatively kept in‐plane in a slightly distorted octahedral configuration. In addition, the distortion angle of Fe, induced by the front‐side species, is largely remedied in the presence of the opposite water (Figure 9c). On the other hand, the adaptive behavior of the backside water molecule varies with different adsorbate species as ORR proceeds, engendering the Fe SA featuring distinct oxidation states (Figure 9d). The electronic structure analysis of the Fe center reveals that the predicted Mulliken spin population is *S* = 2/2 for Fe(IV) in *O…OH^δ−^. This emphasized the appearance of the backside water altering the crystal field of Fe to an octahedral pattern since the square planar one would predict *S* = 0 for Fe(IV). More critically, a novel dissociative ORR mechanism to form “pseudo‐adsorbed” OH species is discovered unprecedently, as aforementioned (Figure 1c,d). In the PCET steps, the proton species (in the form of hydronium in neutral/acidic media or water in an alkaline medium) can protonate the pseudo‐adsorbed hydroxide without traveling to the direct catalyst surface. This, therefore, expands the reactive region beyond the direct catalyst surface, boosting the reaction kinetics via alleviating mass transfer limits (Figure 9e).

The reaction species of OH^−^ in water‐electrolyte can work as a modulator as well. A microkinetic model (MKM) is employed to reveal the self‐adjusting behavior of FeNC and MnNC.^[^ ^148^ ^]^ The active center, Fe site, is covered with the intermediate *OH ranging from 0.28 to 1.00 V (Figure 9f). Distinctively, such *OH becomes part of the active center moiety, Fe(OH)NC, optimizing the electronic structure of SA Fe to boost ORR, exhibiting a self‐adjusting mechanism and predicting a theoretical half‐wave potential of ≈0.88 V. Such intrinsic environmental suitability of SACs demonstrates the necessity of assessing the effect of intrinsic intermediates and the significance of the explicit incorporation of water solvation in the single‐atom electrocatalytic process. The physical orientation of the interfacial water in EDLs during a potential sweep has a significant influence on the electrochemical reactivity of electrode materials.^[^ ^34^, ^155^, ^156^, ^157^ ^]^ By combining in situ Raman spectroscopy and AIMD simulations, three evolutional characteristic water configurations in interfacial region, namely, ‘parallel’, ‘one‐H‐down,’ and ‘two‐H‐down’, have been determined as the electrode potential decreases at electrified Au single‐crystal electrode surfaces, arising to the destruction of the H‐bonds network.^[^ ^34^ ^]^ The statistics of H‐bonds rationalizes the observed blueshift of the O–H stretching mode (ν_O‐H_) in Raman spectra (Figure 9g). Subsequently, the open question here is how the physical orientation of water molecules impacts the electrochemical performance of the electrode materials. Apart from the dynamic coordination behavior of interface water on SA sites and their orientation, the chemisorption of water on metal electrodes is key to differential Helmholtz capacitance.^[^ ^158^ ^]^ AIMD calculations by Le et al. suggest that at more positive potentials, the interface water will adsorb on the surface of Pt(111), which follows the Frumkin adsorption isotherm. The chemisorbed water can induce a significant interface dipole potential and lead to a potential change and, hence, a negative capacitive response. Combining with the normal dielectric response of the solvent, the experimentally observed bell‐shaped differential capacitance of the Helmholtz layer can be reproduced and understood at the molecular level.

### 3.3 Alkali Cation Effects and Interfacial pH

**Figure 10:**
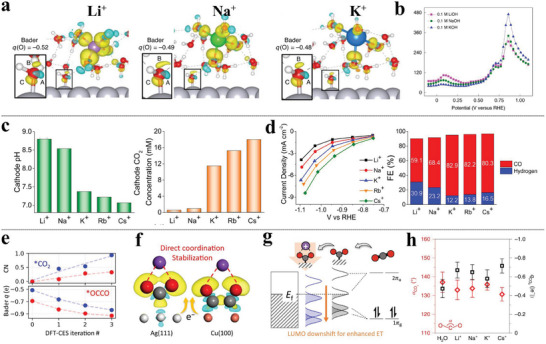
a) Electron density difference maps of the Pt (111)‐OH_ad_‐water interface after introducing Li^+^ (left), Na^+^ (middle), and K^+^ (right). b) Experimental double‐layer capacitance (C_dl_) on the Pt disc electrode at different applied potentials in 0.1 M MOH solutions (*M*  =  Li, Na, and K). Reproduced with permission.^[^ ^165^ ^]^ Copyright 2022, Springer Nature. c) Calculated values of cathode pH (left), and cathode CO concentration (right) over Ag electrodes at −1.0 V versus RHE in CO_2_‐saturated 0.1 M MHCO_3_ (M = Li, Na, K, Rb, Cs) electrolyte. d) Current densities (left) and FEs of CO and H_2_ (right) versus applied potential on Ag cathode at −1.0 V versus RHE. Reproduced with permission.^[^ ^40^ ^]^ Copyright 2016, American Chemical Society. e) Coordinate number change of K^+^ to the *CO_2_ and *OCCO, and that in the Bader charge (Bader q) of those two intermediates during DFT‐CES iterations at −0.5 V_SHE_. f) Cation coupling to the intermediates g) A schematic showing the energy level change of CO_2_ with and without metal cations, based on the PDOS analyses. Reproduced with permission.^[^ ^160^ ^]^ Copyright 2022, Springer Nature. h) OCO angle in CO_2_ and its Bader charge in the presence of different metal cations. Reproduced with permission.^[^ ^161^ ^]^ Copyright 2021, Springer Nature.

Particularly, the presence of alkali metal cations (M^+^) in the interfacial region^[^ ^159^ ^]^ can significantly alter the electrochemical reactivity and selectivity by directly participating in the reaction^[^ ^160^, ^161^ ^]^ or by regulating the local electric field, as suggested by Chan group,^[^ ^39^, ^162^, ^163^, ^164^ ^]^ or by tuning local pH^[^ ^40^ ^]^ to modify the kinetics. Conventionally, the concentration of cations is determined by their size, in which smaller sizes lead to higher local concentrations, resulting in a greater accumulated surface charge and the subsequent interfacial field under a certain potential. With an exception of the hydroxyl adsorbate on the platinum surface in the process of HER, DFT calculations have evidenced that with the presence of alkali cations, the surface electron redistribution locates between the adsorbates *OH and the nearby water molecules, instead of the Pt surface, and follows the trend of Li^+^ > Na^+^ > K^+^ (**Figure** **10**a).^[^ ^165^ ^]^ The induced stronger local electric field from its higher charge density in Li^+^ solutions is responsible for the more distinct charge redistribution. Briefly, the C_dl_ is limited by the potential‐induced *OH surface coverage and *OH adsorption strength. At a higher potential regime (> 0.60 V_RHE_), where there are rich *OH species on Pt, the hydration‐sphere size and the related cation‐surface distance play a critical role in the capacitance (C_dl_ (K^+^) > C_dl_ (Na^+^) > C_dl_ (Li^+^) while at lower potential region (< 0.60 V_RHE_), where there is only a limited *OH coverage, the capacitance (Li^+^ > Na^+^ > K^+^) is more dictated by the local cation concentration, echoed with the common sense (Figure 10b). Generally, cations from the electrolyte serve as counterions to balance the charge of the negatively charged electrode.^[^ ^14^, ^88^ ^]^ However, the direct adsorption of alkali cations on the electrocatalyst of one‐dimensional cobalt‐dithiolene metal‐organic frameworks (MOF) is worth our attention.^[^ ^166^ ^]^ DFT calculations have demonstrated that the hydration energy of the electronegative Na^+^ is much higher than that of the adsorption energy on the bridge‐sites between the two S atoms. The preadsorbed Na^+^ induces the charge redistribution between the S atoms, which has a profound effect on the ΔG_*H_ values. Li with higher electronegativity than that of Na and K has the lowest ΔG_*H_.

On the other hand, the local interfacial pH is an echo of cation identify (Figure 10c).^[^ ^40^ ^]^ Experimental investigations show the interfacial pH decreases ≈9 to ≈7 with increasing cation radius from Li^+^ to Cs^+^ over Ag and Cu electrodes, accounting for the buffering capability and the relevant electrochemical CO_2_RR activity of Li^+^ < Na^+^ < K^+^ < Cs^+^ in basic conditions, corroborated Zhang et al. observation of the interfacial pH trend in a bicarbonate electrolyte in CO_2_RR by utilizing a rotating ring disc electrode.^[^ ^167^ ^]^ However, they claim that the CO_2_RR to CO activity remains unperturbed as local pH varies without further elaboration. As perspectives vary, no unanimous conclusion can be drawn currently. This dilemma in pH perspective by cations calls for a comprehensive investigation of the cation status in the electrochemical process (Figure 10d).

It is dictated that metal cations stabilize certain CO_2_RR intermediates or intimately contact the adsorbates through local electrostatic interactions within the electrical double layer.^[^ ^39^, ^160^, ^161^, ^163^ ^]^ Specifically, Shin et al. focused on the alkali cations’ coordinating ability to all possible intermediates in CO_2_RR on Au(111) and Cu(100) electrodes, empowered by DFT in classical explicit solvent (DFT‐CES) methods, and proposed a cation coupled electron transfer (CCET) based mechanism for CO_2_RR, in which K^+^ assists the electron transfer between the electrode and the adsorbed species.^[^ ^160^ ^]^ The increased coordination number of K^+^ to *CO_2_ and *OCCO and the decrease in the intermediates Bader partial charges during iterations suggest a direct coordination stabilization (Figure 10e). Schematic projected density of states (PDOS) shows a downshift of the *CO_2_ LUMO after the cation‐coupling. Also, the Koper group manifests the obligated mechanistic role of M^+^ in CO_2_RR to CO, making them propose an M^+^‐coupling to *CO_2_^δ−^ mechanism (Figure 10f).^[^ ^161^ ^]^ AIMD modeling reveals that the partially coordinated M^+^ with surrounding waters stabilize the *CO_2_^δ−^ intermediate in regards to OCO angle and Bader charge (Figure 10g).

In addition to cations, the anions in acidic media are in fact not innocent to the electrochemical processes. Luo et al. have provided CVs‐based experimental evidence that the stronger interaction of the ClO_4_^−^ with *OH layer leads to slower kinetics of the *OH ↔ *O transition, and a slower ORR rate.^[^ ^168^ ^]^ Based on the observations, they concluded that the rate of the *O↔*OH process acts as a descriptor of the ORR rate to rationalize the different ORR activity trends for stepped Pt surfaces in acidic and alkaline phases. However, the corresponding interfacial mechanism, in which how anions function the ORR reaction, is still vague and calls for an urgent theoretical understanding to clarify microscopic details.

In this section, we demonstrate with examples that the efficacy of contemporary computational techniques in describing the structural and dynamic features of the solid‐liquid interface in electrochemistry. From molecular engineering perspectives, the incorporation of theoretical simulation unveils the identity of the active site of electrocatalysis and constructs a roadmap for structure‐activity relations. The underlying electronic configurations of the active sites and the supporting substrate provide a fundamental understanding for the micro‐/macro‐ electrochemical performance. From the interface‐modeling viewpoint, AIMD simulations verify the catalytic interfaces should be surveyed as an evolving statistical ensemble of multiphases, with a focus on the electrode surface and SA sites, electrolyte water molecules, and reaction species. Additionally, researchers should pay attention to other intricacies involved in the electrochemical courses, such as the distribution of metal cations.

## 4 The Conclusions and Perspectives

Heterogeneous electrocatalysis has emerged as a powerful method in energy conversion processes, with impressive catalytic performance. This review summarizes fundamental concepts in heterogeneous electrocatalysis and detailed thermodynamic and kinetic modeling methods. We also provide a few selected examples from our current research activity, demonstrating how the state‐of‐the‐art AIMD simulations can offer a microscopic understanding of properties at solid‐liquid interfaces and effectively guide experiments to aid in describing the structure and dynamics at interfaces, as well as understanding the microscopic origin of the measured reactivity in experiments. Our contributions underline the significance of considering explicit solvation shells in simulating the electrochemical interface, the new proposed descriptor derived from charge‐dipole interaction to evaluate HER kinetics on SA sites, and the electrode potential‐determined reactivity and selectivity on various electrochemical processes and surfaces. Additionally, we systematically emphasize the unique structural and electronic properties of various surfaces, such as the regulation of the coordination environment of active sites and the corresponding electronic configuration of the active center, aiming to provide essential guidelines for screening and designing electrocatalysts at the atomic scale. We focus on the dynamism of interfaces, including the leaching of SA sites, surface restructuring of metal electrodes under opening conditions, and the response behaviors of the electrolyte molecules to the applied potential, as well as the roles of alkali metals and interfacial pH effects on reaction kinetics. We conclude that theoretical modeling can lead to a new understanding of the electrocatalytic interface and more efficient interfacial design. In addition to the general challenges of making AIMD techniques more accurate and efficient, which are common to other fields, such as solid‐state physics or computational biochemistry, we would like to discuss a few specific points that we believe are specific to solid‐liquid interfaces.

Despite significant progress in modeling solid‐liquid interfaces, several challenges still need to be addressed to develop more realistic and predictive models. One of the primary challenges we foresee is the careful identification of the genuine active center in heterogeneous electrocatalysis. The support phase plays a crucial role in this regard. Theoretical observations suggest that active sites may exist in the adjacent C site of metal Ni in NiN_1_, NiN_2,_ and NiN_3_ electrodes in the Volmer reactions.^[^ ^88^ ^]^ Furthermore, the direct participation of W sites in adsorbing H on Pt_ads_@WC_X_ alerts us to the impact of the substrate phase in the electrochemical process.^[^ ^120^ ^]^ It is crucial to develop substrate materials with large specific areas, good wetting abilities, and durable mesoporous structures to enhance the performance and applicability of the active phase. However, equal attention should be paid to the role of supports as to the supported active center at the interfacial region.

Another challenge in modeling solid‐liquid interfaces is that the pre‐existing mechanisms and descriptors cannot be generalized for all electrode surfaces. We should not apply mechanically to the evolving electrochemical solid‐liquid interface. New reaction pathways could be observed when taking comprehensively into consideration of various factors in simulating the interface. Such as the fully explicit model systems help us to identify the “pseudo‐adsorbed” hydroxide species in ORR,^[^ ^13^ ^]^ dual‐site participation on NiNC rationalize the observed lower overpotential than that of FeNC and CoNC in OER,^[^ ^12^ ^]^ the change of local coordination and position of SA sites in MNC helps the formation of a stable dihydride or dihydrogen intermediate (HMH) in HER.^[^ ^81^ ^]^ In addition, in view of the screening of the electrocatalysts, the descriptors established on bulk metal electrodes could be less effective for SA sites. The traditional volcano plots have been demonstrated to fail to describe the Volmer reaction on MNC due to the strong charge‐dipole interaction between *H and with surrounding water molecules.^[^ ^81^, ^85^ ^]^ The oversimplicity of the capacitor models for 2D materials in the presence of polarizable implicit solvation in conventional CPM, however, arises non‐negligible deviations.^[^ ^52^ ^]^

The stability of the electrocatalysts is a significant concern in the interface region. As discussed in previous sections, the undercoordination and adaptive bonding with the substrate under electrochemical conditions could engender the SA sites or the metallic surface atoms destabilize and eventually aggregate into larger nanostructures. This will not only affect catalytic activity by favorably binding certain reaction intermediates but also compromises the loading efficiency and purity of SAC, hindering widespread commercial applications. This non‐negligible issue also poses complexities in the atomic‐scale understanding of the interface and limits the rational design of electrocatalysts. Recent grand canonical DFT calculations have demonstrated the existence of an electrochemical potential window within which the aggregated metal target can leach away and the resultant SACs or SCCs are more stable than NPs.^[^ ^169^ ^]^ Here comes the challenge that seeking the balance conditions between maintaining the stability of the electrocatalysts or avoiding the poisoning of the instantaneous active center by reaction species to gain simplified and unambiguous information, and simultaneously maximizing the catalytic power calls for a more realistic description of the catalytic interface.

Although many experimental and theoretical works have demonstrated the different alkali metal cations at the interface facilitate the electrocatalytic processes by modifying the local electric field, buffering the interfacial pH, or stabilizing the reaction species,^[^ ^170^ ^]^ a full understanding of this enhancement effect has been a topic of considerable debate. The potassium cations have been manifested to accelerate the CO desorption and CO_2_ activation by increasing surface charge density^[^ ^159^ ^]^ while CO_2_RR is reported to be completely inhibited in the absence of metal cations in solution.^[^ ^161^ ^]^ Therefore, to more completely understand the descriptor that dictates the electrocatalytic activity, it is essential to uncover how the given cations alter the local chemical environment, i.e., the underlying microscopic structure and evolutional behaviors of the ions at the electrified interface. What's more, to the best of our knowledge, the interfacial structure and electrostatic potential distribution of the condensed cation layer in the EDL are only accessible to state‐of‐the‐art machine learning (ML) due to limitations in spatial and time scales in AIMD, and general design principles are still insufficient.

Regarding computational capability, the combination of DFT and AIMD does help us well interpret the solid‐liquid structures and rationally optimize a predictive model. However, the illimitable chemical space of the configurational and dynamical interface, coupled with the infinite time dimension of molecular dynamics is computationally unattainable with AIMD. Recently, the development of ML potentials has significantly revolutionized atomistic modeling in a more extended dimensionality without compromising the ab initio accuracy. Benefitting from systematic ML accelerated MD (MLMD), accurate and efficient calculation of fundamental thermodynamic factors in electrochemistry, including redox potentials, acidity constants, and explicit solvation‐free energies have been accomplished by the designed automated workflow with several orders of magnitude speedup.^[^ ^171^ ^]^ In this regard, as ML continues to develop, it is bound to play an increasingly indispensable role in electrochemistry and other fields. At the same time, the rapid evolution of advanced electron microscopy methods, such as cryogenic techniques, electron ptychography, and multidimensional real‐space charge‐density imaging, achieving sub‐Angstrom spatial resolution, further complements theoretical techniques.^[^ ^172^, ^173^, ^174^, ^175^ ^]^ The collaboration of theoretical and experimental techniques is an invincible approach for modeling electrocatalysis. However, it is time to retrospect the connections between the experimental measurements and computed parameters, such as the efficiency of the microkinetic model (MKM) to relate inherent energetics of the active sites and experimentally measured electrocatalytic rates,^[^ ^88^ ^]^ to gain reliable interface insights.^[^ ^176^, ^177^ ^]^ What's more, in electrochemistry, the transient capture of the structural evolution during operation highlights the future theoretical direction, such as ML, to interpret complex in situ spectroscopic signals^[^ ^147^ ^]^ while computational high‐throughput sampling of electrocatalytic materials requires a sufficiently large experimental dataset to produce validated predictions.^[^ ^178^ ^]^

As we contribute practical models and methods to probe a more realistic solid‐liquid interface, concurrent challenges are confronting us. Computational electrochemistry holds great potential for advancing our atomistic understanding of electrochemical systems and developing new strategies and materials to address pressing energy and environmental challenges. In this review, we have presented current theoretical endeavors, opening challenges, and promising future directions in exploring the solid‐liquid interface in electrochemistry. We anticipate this thematic article would inspire new research enthusiasm.

## Conflict of Interest

The authors declare no conflict of interest.

## References

[1] E. E. Benson , C. P. Kubiak , A. J. Sathrum , J. M. Smieja , Chem. Soc. Rev. 2009, 38, 89. 19088968 10.1039/b804323j

[2] Y. Y. Birdja , E. Perez‐Gallent , M. C. Figueiredo , A. J. Gottle , F. Calle‐Vallejo , M. T. M. Koper , Nat. Energy 2019, 4, 732.

[3] Q. Q. Zhang , J. Q. Guan , Adv. Funct. Mater. 2020, 30, 2000768.

[4] N. M. Adli , W. T. Shan , S. Hwang , W. Samarakoon , S. Karakalos , Y. Li , D. A. Cullen , D. Su , Z. X. Feng , G. F. Wang , G. Wu , Angew. Chem., Int. Ed. 2021, 60, 1022. 10.1002/anie.202012329 33002266

[5] C. M. Zhao , X. Y. Dai , T. Yao , W. X. Chen , X. Q. Wang , J. Wang , J. Yang , S. Q. Wei , Y. E. Wu , Y. D. Li , J. Am. Chem. Soc. 2017, 139, 8078. 28595012 10.1021/jacs.7b02736

[6] C. C. Yan , H. B. Li , Y. F. Ye , H. H. Wu , F. Cai , R. Si , J. P. Xiao , S. Miao , S. H. Xie , F. Yang , Y. S. Li , G. X. Wang , X. H. Bao , Environ. Sci. 2018, 11, 1204.

[7] P. Yan , J. Liu , S. D. Yuan , Y. J. Liu , W. L. Cen , Y. Q. Chen , Appl. Surf. Sci. 2018, 445, 398.

[8] G. L. Chai , K. P. Qiu , M. Qiao , M. M. Titirici , C. X. Shang , Z. X. Guo , Environ. Sci. 2017, 10, 1186.

[9] L. C. Bai , C. S. Hsu , D. T. L. Alexander , H. M. Chen , X. L. Hu , J. Am. Chem. Soc. 2019, 141, 14190. 31418268 10.1021/jacs.9b05268

[10] J. Yang , H. F. Qi , A. Q. Li , X. Y. Liu , X. F. Yang , S. X. Zhang , Q. Zhao , Q. K. Jiang , Y. Su , L. L. Zhang , J. F. Li , Z. Q. Tian , W. Liu , A. Q. Wang , T. Zhang , J. Am. Chem. Soc. 2022, 144, 12062. 35766932 10.1021/jacs.2c02262

[11] H. L. Fei , J. C. Dong , M. J. Arellano‐Jimenez , G. L. Ye , N. D. Kim , E. L. G. Samuel , Z. W. Peng , Z. Zhu , F. Qin , J. M. Bao , M. J. Yacaman , P. M. Ajayan , D. L. Chen , J. M. Tour , Nat. Commun. 2015, 6, 8668. 26487368 10.1038/ncomms9668 PMC4639894

[12] H. L. Fei , J. C. Dong , Y. X. Feng , C. S. Allen , C. Z. Wan , B. Volosskiy , M. F. Li , Z. P. Zhao , Y. L. Wang , H. T. Sun , P. F. An , W. X. Chen , Z. Y. Guo , C. Lee , D. L. Chen , I. Shakir , M. J. Liu , T. D. Hu , Y. D. Li , A. I. Kirkland , X. F. Duan , Y. Huang , Nat. Catal. 2018, 1, 63.

[13] J. W. Chen , Z. S. Zhang , H. M. Yan , G. J. Xia , H. Cao , Y. G. Wang , Nat. Commun. 2022, 13, 1734. 35365615 10.1038/s41467-022-29357-7 PMC8975818

[14] H. Cao , Z. S. Zhang , J. W. Chen , Y. G. Wang , ACS Catal. 2022, 12, 6606.

[15] K. Asakura , H. Nagahiro , N. Ichikuni , Y. Iwasawa , Appl. Catal., A Gen. 1999, 188, 313.

[16] B. T. Qiao , A. Q. Wang , X. F. Yang , L. F. Allard , Z. Jiang , Y. T. Cui , J. Y. Liu , J. Li , T. Zhang , Nat. Chem. 2011, 3, 634. 21778984 10.1038/nchem.1095

[17] Y. G. Wang , D. C. Cantu , M. S. Lee , J. Li , V. A. Glezakou , R. Rousseau , J. Am. Chem. Soc. 2016, 138, 10467. 27480512 10.1021/jacs.6b04187

[18] Y. G. Wang , D. H. Mei , V. A. Glezakou , J. Li , R. Rousseau , Nat. Commun. 2015, 6, 6511. 25735407 10.1038/ncomms7511 PMC4366521

[19] J. C. Liu , Y. Tang , Y. G. Wang , T. Zhang , J. Li , Natl Sci Rev 2018, 5, 638.

[20] X. F. Yang , A. Q. Wang , B. T. Qiao , J. Li , J. Y. Liu , T. Zhang , Acc. Chem. Res. 2013, 46, 1740. 23815772 10.1021/ar300361m

[21] J. Y. Liu , ACS Catal. 2017, 7, 34. 10.1021/acscatal.7b00712 PMC5557611 28824819

[22] A. Q. Wang , J. Li , T. Zhang , Nat. Rev. Chem. 2018, 2, 65.

[23] L. C. Liu , A. Corma , Chem. Rev. 2018, 118, 4981. 29658707 10.1021/acs.chemrev.7b00776 PMC6061779

[24] B. Zandkarimi , A. N. Alexandrova , Wires Comput. Mol. Sci. 2019, 9, e1420.

[25] C. Y. Dong , Y. L. Li , D. Y. Cheng , M. T. Zhang , J. J. Liu , Y. G. Wang , D. Q. Xiao , D. Ma , ACS Catal. 2020, 10, 11011.

[26] Y. S. Wei , L. M. Sun , M. Wang , J. H. Hong , L. L. Zou , H. W. Liu , Y. Wang , M. Zhang , Z. Liu , Y. W. Li , S. Horike , K. Suenaga , Q. Xu , Angew. Chem. Int. Ed. 2020, 59, 16013. 10.1002/anie.202007221 32568423

[27] J. Wang , R. You , C. Zhao , W. Zhang , W. Liu , X. P. Fu , Y. Y. Li , F. Y. Zhou , X. S. Zheng , Q. Xu , T. Yao , C. J. Jia , Y. G. Wang , W. X. Huang , Y. E. Wu , ACS Catal. 2020, 10, 2754.

[28] C. W. Ye , L. Xu , J. Mater. Chem. A 2021, 9, 22218.

[29] Z. P. Zeng , L. Y. Gan , H. B. Yang , X. Z. Su , J. J. Gao , W. Liu , H. Matsumoto , J. Gong , J. M. Zhang , W. Z. Cai , Z. Y. Zhang , Y. B. Yan , B. Liu , P. Chen , Nat. Commun. 2021, 12, 4088. 34215728 10.1038/s41467-021-24052-5 PMC8253796

[30] S. Nitopi , E. Bertheussen , S. B. Scott , X. Y. Liu , A. K. Engstfeld , S. Horch , B. Seger , I. E. L. Stephens , K. Chan , C. Hahn , J. K. Norskov , T. F. Jaramillo , I. Chorkendorff , Chem. Rev. 2019, 119, 7610. 31117420 10.1021/acs.chemrev.8b00705

[31] O. M. Magnussen , A. Gross , J. Am. Chem. Soc. 2019, 141, 4777. 30768905 10.1021/jacs.8b13188

[32] X. H. Zhao , Y. Y. Liu , J. Am. Chem. Soc. 2020, 142, 5773. 32122132 10.1021/jacs.9b13872

[33] J. Yang , W. G. Liu , M. Q. Xu , X. Y. Liu , H. F. Qi , L. L. Zhang , X. F. Yang , S. S. Niu , D. Zhou , Y. F. Liu , Y. Su , J. F. Li , Z. Q. Tian , W. Zhou , A. Q. Wang , T. Zhang , J. Am. Chem. Soc. 2021, 143, 14530. 34464109 10.1021/jacs.1c03788

[34] C. Y. Li , J. B. Le , Y. H. Wang , S. Chen , Z. L. Yang , J. F. Li , J. Cheng , Z. Q. Tian , Nat. Mater. 2019, 18, 697. 31036960 10.1038/s41563-019-0356-x

[35] Y. H. Liu , T. Kawaguchi , M. S. Pierce , V. Komanicky , H. You , J. Phys. Chem. Lett. 2018, 9, 1265. 29481094 10.1021/acs.jpclett.8b00123

[36] Q. S. Zhu , S. K. Wallentine , G. H. Deng , J. A. Rebstock , L. R. Baker , JACS Au 2022, 2, 472. 35252996 10.1021/jacsau.1c00512 PMC8889607

[37] M. F. Delley , E. M. Nichols , J. M. Mayer , J. Phys. Chem. C 2022, 126, 8477. 10.1021/acs.jpcc.2c01134 PMC12306796 40735495

[38] V. J. Ovalle , Y. S. Hsu , N. Agrawal , M. J. Janik , M. M. Waegele , Nat. Catal. 2022, 5, 624.

[39] J. Resasco , L. D. Chen , E. Clark , C. Tsai , C. Hahn , T. F. Jaramillo , K. Chan , A. T. Bell , J. Am. Chem. Soc. 2017, 139, 11277. 28738673 10.1021/jacs.7b06765

[40] M. R. Singh , Y. Kwon , Y. Lum , J. W. Ager , A. T. Bell , J. Am. Chem. Soc. 2016, 138, 13006. 27626299 10.1021/jacs.6b07612

[41] J. Liu , Chin. J. Catal. 2017, 38, 1460.

[42] C. Asokan , L. DeRita , P. Christopher , Chin. J. Catal. 2017, 38, 1473.

[43] B. Garlyyev , S. Xue , S. Watzele , D. Scieszka , A. S. Bandarenka , J. Phys. Chem. Lett. 2018, 9, 1927. 29595987 10.1021/acs.jpclett.8b00610

[44] J. J. Velasco‐Velez , C. H. Wu , T. A. Pascal , L. W. F. Wan , J. H. Guo , D. Prendergast , M. Salmeron , Science 2014, 346, 831. 25342657 10.1126/science.1259437

[45] M. F. Toney , J. N. Howard , J. Richer , G. L. Borges , J. G. Gordon , O. R. Melroy , D. G. Wiesler , D. Yee , L. B. Sorensen , Nature 1994, 368, 444.

[46] S. Q. Zhu , X. P. Qin , Y. Yao , M. H. Shao , J. Am. Chem. Soc. 2020, 142, 8748. 32306730 10.1021/jacs.0c01104

[47] Z. W. Seh , J. Kibsgaard , C. F. Dickens , I. B. Chorkendorff , J. K. Norskov , T. F. Jaramillo , Science 2017, 355, eaad4998. 28082532 10.1126/science.aad4998

[48] D. Bedrov , J. P. Piquemal , O. Borodin , A. D. MacKerell , B. Roux , C. Schroder , Chem. Rev. 2019, 119, 7940. 31141351 10.1021/acs.chemrev.8b00763 PMC6620131

[49] S. J. Marrink , H. J. Risselada , S. Yefimov , D. P. Tieleman , A. H. de Vries , J. Phys. Chem. B 2007, 111, 7812. 17569554 10.1021/jp071097f

[50] L. L. Li , X. Chang , X. Y. Lin , Z. J. Zhao , J. L. Gong , Chem. Soc. Rev. 2020, 49, 8156. 32870221 10.1039/d0cs00795a

[51] J. B. Le , X. H. Yang , Y. B. Zhuang , M. Jia , J. Cheng , J. Phys. Chem. Lett. 2021, 12, 8924. 34499508 10.1021/acs.jpclett.1c02086

[52] J. A. Gauthier , S. Ringe , C. F. Dickens , A. J. Garza , A. T. Bell , M. Head‐Gordon , J. K. Norskov , K. R. Chan , ACS Catal. 2019, 9, 920.

[53] J. K. Norskov , J. Rossmeisl , A. Logadottir , L. Lindqvist , J. R. Kitchin , T. Bligaard , H. Jonsson , J. Phys. Chem. B 2004, 108, 17886. 10.1021/jp047349j 39682080

[54] J. H. Montoya , C. Shi , K. Chan , J. K. Norskov , J. Phys. Chem. Lett. 2015, 6, 2032. 26266498 10.1021/acs.jpclett.5b00722

[55] X. W. Nie , M. R. Esopi , M. J. Janik , A. Asthagiri , Angew. Chem., Int. Ed. 2013, 52, 2459. 10.1002/anie.201208320 23345201

[56] H. H. Heenen , J. A. Gauthier , H. H. Kristoffersen , T. Ludwig , K. Chan , J. Chem. Phys. 2020, 152, 144703. 32295363 10.1063/1.5144912

[57] H. Cao , G. J. Xia , J. W. Chen , H. M. Yan , Z. Huang , Y. G. Wang , J. Phys. Chem. C 2020, 124, 7287.

[58] H. M. Yan , Z. X. Wang , Y. M. Wang , G. J. Xia , Y. G. Wang , J. Phys. Chem. C 2022, 126, 7841.

[59] S. J. Qian , H. Cao , J. W. Chen , J. C. Chen , Y. G. Wang , J. Li , ACS Catal. 2022, 12, 11530.

[60] R. Sundararaman , W. A. Goddard , J. Chem. Phys. 2015, 142, 064107. 25681887 10.1063/1.4907731

[61] F. Abild‐Pedersen , J. Greeley , F. Studt , J. Rossmeisl , T. R. Munter , P. G. Moses , E. Skulason , T. Bligaard , J. K. Norskov , Phys. Rev. Lett. 2007, 99, 016105. 17678168 10.1103/PhysRevLett.99.016105

[62] H. L. Xin , A. Vojvodic , J. Voss , J. K. Norskov , F. Abild‐Pedersen , Phys. Rev. B 2014, 89, 115114.

[63] E. M. Fernandez , P. G. Moses , A. Toftelund , H. A. Hansen , J. I. Martinez , F. Abild‐Pedersen , J. Kleis , B. Hinnemann , J. Rossmeisl , T. Bligaard , J. K. Norskov , Angew. Chem., Int. Ed. 2008, 47, 4683. 10.1002/anie.200705739 18484577

[64] M. Busch , M. D. Wodrich , C. Corminboeuf , Chem. Sci. 2015, 6, 6754. 28757966 10.1039/c5sc02910d PMC5508671

[65] J. Greeley , Annu. Rev. Chem. Biomol. Eng. 2016, 7, 605. 27088666 10.1146/annurev-chembioeng-080615-034413

[66] J. C. Jiang , J. C. Chen , M. D. Zhao , Q. Yu , Y. G. Wang , J. Li , Nano Res. 2022, 15, 7116.

[67] H. X. Xu , D. J. Cheng , D. P. Cao , X. C. Zeng , Nat. Catal. 2018, 1, 339.

[68] Y. Jiao , Y. Zheng , M. Jaroniec , S. Z. Qiao , J. Am. Chem. Soc. 2014, 136, 4394. 24580116 10.1021/ja500432h PMC3986026

[69] J. Y. Cheon , J. H. Kim , J. H. Kim , K. C. Goddeti , J. Y. Park , S. H. Joo , J. Am. Chem. Soc. 2014, 136, 8875. 24911055 10.1021/ja503557x

[70] G. Di Liberto , L. A. Cipriano , G. Pacchioni , ACS Catal. 2022, 12, 5846.

[71] K. S. Exner , Int J Hydrogen Energy 2020, 45, 27221.

[72] P. Lindgren , G. Kastlunger , A. A. Peterson , ACS Catal. 2020, 10, 121.

[73] H. Ooka , R. Nakamura , J. Phys. Chem. Lett. 2019, 10, 6706. 31625745 10.1021/acs.jpclett.9b01796

[74] F. Calle‐Vallejo , M. T. M. Koper , Angew. Chem., Int. Ed. 2013, 52, 7282. 10.1002/anie.201301470 23733719

[75] J. C. Chen , H. Cao , J. W. Chen , S. J. Qian , G. J. Xia , Y. G. Wang , J. Li , J. Phys. Chem. C 2021, 125, 19821.

[76] T. Sours , A. Patel , J. Norskov , S. Siahrostami , A. Kulkarni , J. Phys. Chem. Lett. 2020, 11, 10029. 33179928 10.1021/acs.jpclett.0c02889

[77] M. J. Craig , G. Coulter , E. Dolan , J. Soriano‐Lopez , E. Mates‐Torres , W. Schmitt , M. Garcia‐Melchor , Nat. Commun. 2019, 10, 4993. 31704927 10.1038/s41467-019-12994-w PMC6841662

[78] W. Zhang , G. J. Xia , Y. G. Wang , Chin. J. Catal. 2022, 43, 167.

[79] X. P. Gao , L. Mei , Y. N. Zhou , Z. M. Shen , Nanoscale 2020, 12, 7814. 32219292 10.1039/c9nr10579d

[80] Y. W. Li , Q. Sun , Adv. Energy Mater. 2016, 6, 1600463.

[81] G. Di Liberto , L. A. Cipriano , G. Pacchioni , J. Am. Chem. Soc. 2021, 143, 20431. 34821146 10.1021/jacs.1c10470 PMC8662730

[82] J. K. Norskov , T. Bligaard , A. Logadottir , J. R. Kitchin , J. G. Chen , S. Pandelov , J. K. Norskov , J. Electrochem. Soc. 2005, 152, J23.

[83] M. D. Hossain , Z. J. Liu , M. H. Zhuang , X. X. Yan , G. L. Xu , C. A. Gadre , A. Tyagi , I. H. Abidi , C. J. Sun , O. L. Wong , A. Guda , Y. F. Hao , X. Q. Pan , K. Amine , Z. T. Luo , Adv. Energy Mater. 2019, 9, 1803689.

[84] H. Y. Li , G. L. Wang , F. R. Zhang , L. L. Zou , Z. Q. Zou , H. Yang , J. Phys. Chem. C 2020, 124, 11760.

[85] H. Cao , Q. L. Wang , Z. S. Zhang , H. M. Yan , H. Y. Zhao , H. B. Yang , B. Liu , J. Li , Y. G. Wang , J. Am. Chem. Soc. 2023, 145, 13038. 37285479 10.1021/jacs.2c13418

[86] B. Z. Lu , Q. M. Liu , S. W. Chen , ACS Catal. 2020, 10, 7584.

[87] T. Cheng , H. Xiao , W. A. Goddard , J. Phys. Chem. Lett. 2015, 6, 4767. 26562750 10.1021/acs.jpclett.5b02247

[88] H. Y. Zhao , H. Cao , Z. S. Zhang , Y. G. Wang , ACS Catal. 2022, 12, 11380.

[89] T. Cheng , H. Xiao , W. A. Goddard , J. Am. Chem. Soc. 2016, 138, 13802. 27726392 10.1021/jacs.6b08534

[90] S. Ringe , C. G. Morales‐Guio , L. D. Chen , M. Fields , T. F. Jaramillo , C. Hahn , K. Chan , Nat. Commun. 2020, 11, 33. 31911585 10.1038/s41467-019-13777-z PMC6946669

[91] A. J. Gottle , M. T. M. Koper , Chem. Sci. 2017, 8, 458. 28451193 10.1039/c6sc02984a PMC5298188

[92] H. A. Hansen , J. Rossmeisl , J. K. Norskov , Phys. Chem. Chem. Phys. 2008, 10, 3722. 18563233 10.1039/b803956a

[93] S. Vijay , W. Ju , S. Bruckner , S. C. Tsang , P. Strasser , K. R. Chan , Nat. Catal. 2021, 4, 1024.

[94] K. R. Chan , J. K. Norskov , J. Phys. Chem. Lett. 2015, 6, 2663. 26266844 10.1021/acs.jpclett.5b01043

[95] J. Hussain , H. Jonsson , E. Skulason , ACS Catal. 2018, 8, 5240.

[96] X. H. Zhao , Y. Y. Liu , J. Am. Chem. Soc. 2021, 143, 9423. 10.1021/jacs.1c02186 34133170

[97] W. Schmickler , E. Santos , Interfacial Electrochemistry, 2ed. Springer, Berlin 2010.

[98] J. S. Filhol , M. Neurock , Angew. Chem., Int Ed. 2006, 45, 402. 10.1002/anie.200502540 16307461

[99] L. Yu , X. L. Pan , X. M. Cao , P. Hu , X. H. Bao , J. Catal. 2011, 282, 183.

[100] T. Cheng , L. Wang , B. V. Merinov , W. A. Goddard , J. Am. Chem. Soc. 2018, 140, 7787. 29792321 10.1021/jacs.8b04006

[101] J. J. Li , J. Liu , B. Yang , J. Energy Chem. 2021, 53, 20.

[102] M. D. Hossain , Y. F. Huang , T. H. Yu , W. A. Goddard , Z. T. Luo , Nat. Commun. 2020, 11, 2256. 32382033 10.1038/s41467-020-16119-6 PMC7205999

[103] Y. F. Huang , R. J. Nielsen , W. A. Goddard , J. Am. Chem. Soc. 2018, 140, 16773. 30406657 10.1021/jacs.8b10016

[104] Y. Wang , Numerical Simulation of Electrified Solid–Liquid Interfaces, AIP Publishing, Melville, New York 2021.

[105] H. Xiao , T. Cheng , W. A. Goddard , R. Sundararaman , J. Am. Chem. Soc. 2016, 138, 483. 26716884 10.1021/jacs.5b11390

[106] R. Sundararaman , K. Letchworth‐Weaver , K. A. Schwarz , D. Gunceler , Y. Ozhabes , T. A. Arias , SoftwareX 2017, 6, 278. 29892692 10.1016/j.softx.2017.10.006 PMC5992620

[107] Z. M. Xia , H. Xiao , J. Chem. Theory Comput. 2023, 19, 5168. 37399292 10.1021/acs.jctc.3c00237

[108] J. Rossmeisl , E. Skulason , M. E. Bjorketun , V. Tripkovic , J. K. Norskov , Chem. Phys. Lett. 2008, 466, 68.

[109] K. Chant , J. K. Norskov , J. Phys. Chem. Lett. 2016, 7, 1686. 27088442 10.1021/acs.jpclett.6b00382

[110] J. A. Gauthier , C. F. Dickens , H. H. Heenen , S. Vijay , S. Ringe , K. Chan , J. Chem. Theory Comput. 2019, 15, 6895. 31689089 10.1021/acs.jctc.9b00717

[111] D. Kim , J. J. Shi , Y. Y. Liu , J. Am. Chem. Soc. 2018, 140, 9127. 29956544 10.1021/jacs.8b03002

[112] J. Chen , C. Li , G. Q. Shi , J. Phys. Chem. Lett. 2013, 4, 1244. 26282137 10.1021/jz400160k

[113] J. P. Randin , E. Yeager , J. Electroanal. Chem. 1972, 36, 257.

[114] J. H. Zhong , J. Y. Liu , Q. Y. Li , M. G. Li , Z. C. Zeng , S. Hu , D. Y. Wu , W. W. Cai , B. Ren , Electrochim. Acta 2013, 110, 754.

[115] L. L. Zhang , X. Zhao , H. X. Ji , M. D. Stoller , L. F. Lai , S. Murali , S. Mcdonnell , B. Cleveger , R. M. Wallace , R. S. Ruoff , Environ. Sci. 2012, 5, 9618.

[116] C. Zhan , J. Neal , J. Z. Wu , D. E. Jiang , J. Phys. Chem. C 2015, 119, 22297.

[117] X. W. Bai , X. H. Zhao , Y. H. Zhang , C. Y. Ling , Y. P. Zhou , J. L. Wang , Y. Y. Liu , J. Am. Chem. Soc. 2022, 144, 17140. 36089737 10.1021/jacs.2c07178

[118] L. L. Patera , F. Bianchini , C. Africh , C. Dri , G. Soldano , M. M. Mariscal , M. Peressi , G. Comelli , Science 2018, 359, 1243. 29590072 10.1126/science.aan8782

[119] X. Su , X. F. Yang , Y. Q. Huang , B. Liu , T. Zhang , Acc. Chem. Res. 2019, 52, 656. 30512920 10.1021/acs.accounts.8b00478

[120] T. Ma , H. Cao , S. Li , S. J. Cao , Z. Y. Zhao , Z. H. Wu , R. Yan , C. D. Yang , Y. Wang , P. A. van Aken , L. Qiu , Y. G. Wang , C. Cheng , Adv. Mater. 2022, 34, 2206368. 10.1002/adma.202206368 35987876

[121] T. T. Sun , Y. L. Li , T. T. Cui , L. B. Xu , Y. G. Wang , W. X. Chen , P. P. Zhang , T. Y. Zheng , X. Z. Fu , S. L. Zhang , Z. D. Zhang , D. S. Wang , Y. D. Li , Nano Lett. 2020, 20, 6206. 32697097 10.1021/acs.nanolett.0c02677

[122] M. Li , H. Y. Zhu , Q. Yuan , T. Y. Li , M. M. Wang , P. Zhang , Y. L. Zhao , D. L. Qin , W. Y. Guo , B. Liu , X. Yang , Y. Q. Liu , Y. Pan , Adv. Funct. Mater. 2023, 33, 2210867.

[123] T. T. Sun , L. B. Xu , D. S. Wang , Y. D. Li , Nano Res. 2019, 12, 2067.

[124] X. Zhang , Z. S. Wu , X. Zhang , L. W. Li , Y. Y. Li , H. M. Xu , X. X. Li , X. L. Yu , Z. S. Zhang , Y. Y. Liang , H. L. Wang , Nat. Commun. 2017, 8, 14675. 28272403 10.1038/ncomms14675 PMC5344970

[125] X. Zhang , Y. Wang , M. Gu , M. Y. Wang , Z. S. Zhang , W. Y. Pan , Z. Jiang , H. Z. Zheng , M. Lucero , H. L. Wang , G. E. Sterbinsky , Q. Ma , Y. G. Wang , Z. X. Feng , J. Li , H. J. Dai , Y. Y. Liang , Nat. Energy 2020, 5, 684.

[126] Z. S. Zhang , Y. G. Wang , J. Phys. Chem. C 2021, 125, 13836.

[127] D. Cheng , Z. Wei , Z. Zhang , P. Broekmann , Anastassia N. Alexandrova* , P. Sautet* , Angew. Chem., Int. Ed. 2023, 62, e202218575. 10.1002/anie.202218575 36922903

[128] Z. S. Zhang , Z. Y. Wei , P. Sautet , A. N. Alexandrova , J. Am. Chem. Soc. 2022, 144, 19284. 36227161 10.1021/jacs.2c06188

[129] S. B. Han , G. J. Xia , C. Cai , Q. Wang , Y. G. Wang , M. Gu , J. Li , Nat. Commun. 2020, 11, 552. 31992711 10.1038/s41467-019-14212-z PMC6987310

[130] X. Y. Li , H. P. Rong , J. T. Zhang , D. S. Wang , Y. D. Li , Nano Res. 2020, 13, 1842.

[131] W. G. Liu , L. L. Zhang , X. Liu , X. Y. Liu , X. F. Yang , S. Miao , W. T. Wang , A. Q. Wang , T. Zhang , J. Am. Chem. Soc. 2017, 139, 10790. 28745500 10.1021/jacs.7b05130

[132] X. Q. Wang , Z. Chen , X. Y. Zhao , T. Yao , W. X. Chen , R. You , C. M. Zhao , G. Wu , J. Wang , W. X. Huang , J. L. Yang , X. Hong , S. Q. Wei , Y. Wu , Y. D. Li , Angew. Chem. Int. Ed. 2018, 57, 1944. 10.1002/anie.201712451 29266615

[133] G. K. Han , Y. Zheng , X. Zhang , Z. Q. Wang , Y. Gong , C. Y. Du , M. N. Banis , Y. M. Yiu , T. K. Sham , L. Gu , Y. R. Sun , Y. J. Wang , J. P. Wang , Y. Z. Gao , G. P. Yin , X. L. Sun , Nano Energy 2019, 66, 104088.

[134] H. H. Wu , H. B. Li , X. F. Zhao , Q. F. Liu , J. Wang , J. P. Xiao , S. H. Xie , R. Si , F. Yang , S. Miao , X. G. Guo , G. X. Wang , X. H. Bao , Environ. Sci. 2016, 9, 3736.

[135] N. K. Wagh , S. S. Shinde , C. H. Lee , J. Y. Jung , D. H. Kim , S. H. Kim , C. Lin , S. U. Lee , J. H. Lee , Appl. Catal., B 2020, 268, 118746.

[136] Y. T. Qu , Z. J. Li , W. X. Chen , Y. Lin , T. W. Yuan , Z. K. Yang , C. M. Zhao , J. Wang , C. Zhao , X. Wang , F. Y. Zhou , Z. B. Zhuang , Y. Wu , Y. D. Li , Nat. Catal. 2018, 1, 781.

[137] L. C. Liu , A. Corma , Trends Chem. 2020, 2, 383.

[138] G. A. Somorjai , Annu. Rev. Phys. Chem. 1994, 45, 721.

[139] X. Han , T. Y. Zhang , W. X. Chen , B. Dong , G. Meng , L. R. Zheng , C. Yang , X. M. Sun , Z. B. Zhuang , D. S. Wang , A. J. Han , J. F. Liu , Adv. Energy Mater. 2021, 11, 2002753.

[140] Z. Weng , Y. S. Wu , M. Y. Wang , J. B. Jiang , K. Yang , S. J. Huo , X. F. Wang , Q. Ma , G. W. Brudvig , V. S. Batista , Y. Y. Liang , Z. X. Feng , H. L. Wang , Nat. Commun. 2018, 9, 415. 29379087 10.1038/s41467-018-02819-7 PMC5788987

[141] Q. Jia , N. Ramaswamy , H. Hafiz , U. Tylus , K. Strickland , G. Wu , B. Barbiellini , A. Bansil , E. F. Holby , P. Zelenay , S. Mukerjee , ACS Nano 2015, 9, 12496. 26566192 10.1021/acsnano.5b05984

[142] Q. Hu , Z. Han , X. D. Wang , G. M. Li , Z. Y. Wang , X. W. Huang , H. P. Yang , X. Z. Ren , Q. L. Zhang , J. H. Liu , C. X. He , Angew. Chem., Int. Ed. 2020, 59, 19054. 10.1002/anie.202009277 32686303

[143] D. H. Lim , J. H. Jo , D. Y. Shin , J. Wilcox , H. C. Ham , S. W. Nam , Nanoscale 2014, 6, 5087. 24695587 10.1039/c3nr06539a

[144] D. Karapinar , N. T. Huan , N. R. Sahraie , J. K. Li , D. Wakerley , N. Touati , S. Zanna , D. Taverna , L. H. G. Tizei , A. Zitolo , F. Jaouen , V. Mougel , M. Fontecave , Angew Chem Int Edit 2019, 58, 15098. 10.1002/anie.201907994 31453650

[145] D. Karapinar , A. Zitolo , T. N. Huan , S. Zanna , D. Taverna , L. H. G. Tizei , D. Giaume , P. Marcus , V. Mougel , M. Fontecave , ChemSusChem 2020, 13, 173. 31622012 10.1002/cssc.201902859

[146] A. S. Varela , W. Ju , A. Bagger , P. Franco , J. Rossmeisl , P. Strasser , ACS Catal. 2019, 9, 7270.

[147] H. Su , W. L. Zhou , H. Zhang , W. Zhou , X. Zhao , Y. L. Li , M. H. Liu , W. R. Cheng , Q. H. Liu , J. Am. Chem. Soc. 2020, 142, 12306. 32579351 10.1021/jacs.0c04231

[148] Y. Wang , Y. J. Tang , K. Zhou , J. Am. Chem. Soc. 2019, 141, 14115. 31468961 10.1021/jacs.9b07712

[149] J. F. Huang , N. Hormann , E. Oveisi , A. Loiudice , G. L. De Gregorio , O. Andreussi , N. Marzari , R. Buonsanti , Nat. Commun. 2018, 9, 3117. 30082872 10.1038/s41467-018-05544-3 PMC6079067

[150] B. Eren , D. Zherebetskyy , L. L. Patera , C. H. Wu , H. Bluhm , C. Africh , L. W. Wang , G. A. Somorjai , M. Salmeron , Science 2016, 351, 475. 26823421 10.1126/science.aad8868

[151] Y. G. Kim , J. H. Baricuatro , A. Javier , J. M. Gregoire , M. P. Soriaga , Langmuir 2014, 30, 15053. 25489793 10.1021/la504445g

[152] A. Auer , M. Andersen , E. M. Wernig , N. G. Hormann , N. Buller , K. Reuter , J. Kunze‐Liebhauser , Nat. Catal. 2020, 3, 797.

[153] M. S. Hofman , D. Z. Wang , Y. X. Yang , B. E. Koel , Surf. Sci. Rep. 2018, 73, 153.

[154] E. F. Holby , G. F. Wang , P. Zelenay , ACS Catal. 2020, 10, 14527.

[155] L. F. Scatena , M. G. Brown , G. L. Richmond , Science 2001, 292, 908. 11340199 10.1126/science.1059514

[156] J. Carrasco , A. Hodgson , A. Michaelides , Nat. Mater. 2012, 11, 667. 22825022 10.1038/nmat3354

[157] S. Gim , K. J. Cho , H. K. Lim , H. Kim , Stem Cells Int. 2019, 9, 14805./bib>

[158] J. B. Le , Q. Y. Fan , J. Q. Li , J. Cheng , Sci. Adv. 2020, 6, eabb1219. 33028519 10.1126/sciadv.abb1219 PMC7541063

[159] S. Chandrashekar , H. P. I. van Montfort , D. Bohra , G. Filonenko , H. Geerlings , T. Burdyny , W. A. Smith , Nanoscale 2022, 14, 14185. 36124967 10.1039/d2nr03438g

[160] S. J. Shin , H. Choi , S. Ringe , D. H. Won , H. S. Oh , D. H. Kim , T. Lee , D. H. Nam , H. Kim , C. H. Choi , Nat. Commun. 2022, 13, 5482. 36123326 10.1038/s41467-022-33199-8 PMC9485141

[161] M. C. O. Monteiro , F. Dattila , B. Hagedoorn , R. Garcia‐Muelas , N. Lopez , M. T. M. Koper , Nat. Catal. 2021, 4, 654.

[162] S. Ringe , E. L. Clark , J. Resasco , A. Walton , B. Seger , A. T. Bell , K. Chan , Environ. Sci. 2019, 12, 3001.

[163] L. D. Chen , M. Urushihara , K. R. Chan , J. K. Norskov , ACS Catal. 2016, 6, 7133.

[164] R. B. Sandberg , J. H. Montoya , K. Chan , J. K. Norskov , Surf. Sci. 2016, 654, 56.

[165] A. H. Shah , Z. S. Zhang , Z. H. Huang , S. B. Wang , G. Y. Zhong , C. Z. Wan , A. N. Alexandrova , Y. Huang , X. F. Duan , Nat. Catal. 2022, 5, 923.

[166] Y. Wang , X. Liu , J. X. Liu , M. Al‐Mamun , A. W. C. Liew , H. J. Yin , W. Wen , Y. L. Zhong , P. R. Liu , H. J. Zhao , ACS Appl Energy Mater 2018, 1, 1688.

[167] F. Zhang , A. C. Co , Angew. Chem., Int. Ed. 2020, 59, 1674. 10.1002/anie.201912637 31721382

[168] M. C. Luo , M. T. M. Koper , Nat. Catal. 2022, 5, 615.

[169] J. C. Liu , H. Xiao , J. Li , J. Am. Chem. Soc. 2020, 142, 3375. 31994381 10.1021/jacs.9b06808

[170] M. M. Waegele , C. M. Gunathunge , J. Y. Li , X. Li , J. Chem. Phys. 2019, 151, 160902. 31675864 10.1063/1.5124878

[171] F. Wang , J. Cheng , J. Chem. Phys. 2022, 157, 024103. 35840372 10.1063/5.0098330

[172] Y. Jiang , Z. Chen , Y. M. Hang , P. Deb , H. Gao , S. E. Xie , P. Purohit , M. W. Tate , J. Park , S. M. Gruner , V. Elser , D. A. Muller , Nature 2018, 559, 343. 30022131 10.1038/s41586-018-0298-5

[173] W. P. Gao , C. Addiego , H. Wang , X. X. Yan , Y. S. Hou , D. X. Ji , C. Heikes , Y. Zhang , L. Z. Li , H. X. Huyan , T. Blum , T. Aoki , Y. F. Nie , D. G. Schlom , R. Q. Wu , X. Q. Pan , Nature 2019, 575, 480. 31610544 10.1038/s41586-019-1649-6

[174] Y. B. Li , W. Huang , Y. Z. Li , W. Chiu , Y. Cui , ACS Nano 2020, 14, 9263. 32806083 10.1021/acsnano.0c05020

[175] Z. Chen , M. Odstrcil , Y. Jiang , Y. M. Han , M. H. Chiu , L. J. Li , D. A. Muller , Nat. Commun. 2020, 11, 2994. 32533001 10.1038/s41467-020-16688-6 PMC7293311

[176] S. Bhandari , S. Rangarajan , M. Mavrikakis , Acc. Chem. Res. 2020, 53, 1893. 32869965 10.1021/acs.accounts.0c00340

[177] A. H. Motagamwala , J. A. Dumesic , Chem. Rev. 2021, 121, 1049. 33205961 10.1021/acs.chemrev.0c00394

[178] M. Zhong , K. Tran , Y. M. Min , C. H. Wang , Z. Y. Wang , C. T. Dinh , P. De Luna , Z. Q. Yu , A. S. Rasouli , P. Brodersen , S. Sun , O. Voznyy , C. S. Tan , M. Askerka , F. L. Che , M. Liu , A. Seifitokaldani , Y. J. Pang , S. C. Lo , A. Ip , Z. Ulissi , E. H. Sargent , Nature 2020, 581, 178. 32405017 10.1038/s41586-020-2242-8

